# Social brain activation during mentalizing in a large autism cohort: the Longitudinal European Autism Project

**DOI:** 10.1186/s13229-020-0317-x

**Published:** 2020-02-22

**Authors:** Carolin Moessnang, Sarah Baumeister, Julian Tillmann, David Goyard, Tony Charman, Sara Ambrosino, Simon Baron-Cohen, Christian Beckmann, Sven Bölte, Carsten Bours, Daisy Crawley, Flavio Dell’Acqua, Sarah Durston, Christine Ecker, Vincent Frouin, Hannah Hayward, Rosemary Holt, Mark Johnson, Emily Jones, Meng-Chuan Lai, Michael V. Lombardo, Luke Mason, Marianne Oldenhinkel, Antonio Persico, Antonia San José Cáceres, Will Spooren, Eva Loth, Declan G. M. Murphy, Jan K. Buitelaar, Tobias Banaschewski, Daniel Brandeis, Heike Tost, Andreas Meyer-Lindenberg, the EU-AIMS LEAP group

**Affiliations:** 1 Department of Psychiatry and Psychotherapy, Central Institute of Mental Health, Medical Faculty Mannheim / University of Heidelberg, Mannheim, Germany; 2 Department of Child and Adolescent Psychiatry and Psychotherapy, Central Institute of Mental Health, Medical Faculty Mannheim / University of Heidelberg, Mannheim, Germany; 3 Department of Psychology, Institute of Psychiatry, Psychology & Neuroscience, King’s College London, London, UK; 4 Department of Applied Psychology: Health, Development, Enhancement, and Intervention, University of Vienna, Vienna, Australia; 5 Neurospin Centre CEA, Saclay, Gif sur Yvette, France; 6 Department of Psychiatry, UMC Utrecht Brain Center, Utrecht, The Netherlands; 7 Autism Research Centre, Department of Psychiatry, University of Cambridge, Cambridge, UK; 8 Donders Institute for Brain, Cognition and Behavior, Radboud University, Nijmegen, The Netherlands; 9 Department of Cognitive Neuroscience, Radboud University Medical Center, Nijmegen, The Netherlands; 10 Center of Neurodevelopmental Disorders (KIND), Centre for Psychiatry Research, Department of Women’s and Children’s Health, Karolinska Institutet and Child and Adolescent Psychiatry, Stockholm Health Care Services, Stockholm County Council, Stockholm, Sweden; 11 Curtin Autism Research Group, School of Occupational Therapy, Social Work and Speech Pathology, Curtin University, Perth, Western Australia Australia; 12 Department of Forensic and Neurodevelopmental Sciences, Institute of Psychiatry, Psychology & Neuroscience, King’s College London, London, UK; 13 Sackler Institute for Translational Neurodevelopment, Institute of Psychiatry, Psychology & Neuroscience, King’s College London, London, UK; 14 Department of Child and Adolescent Psychiatry, Psychosomatics and Psychotherapy, University Hospital Frankfurt am Main, Goethe University, Frankfurt, Germany; 15 Centre for Brain and Cognitive Development, Birkbeck, University of London, London, UK; 16 Centre for Addiction and Mental Health and The Hospital for Sick Children, Department of Psychiatry, University of Toronto, Toronto, Canada; 17 Department of Psychiatry, National Taiwan University Hospital and College of Medicine, Taipei, Taiwan; 18 Department of Psychology, University of Cyprus, Nicosia, Cyprus; 19 Child and Adolescent Neuropsychiatry Unit, “Gaetano Martino” University Hospital, University of Messina, Messina, Italy; 20 Mafalda Luce Center for Pervasive Developmental Disorders, University Campus Bio-Medico, Milan, Italy; 21 Roche Pharmaceutical Research and Early Development, NORD Discovery and Translational Area, Roche Innovation Center Basel, Basel, Switzerland; 22 Karakter Child and Adolescent Psychiatry University Centre, Nijmegen, The Netherlands; 23 Neuroscience Center Zurich, University of Zurich and ETH Zurich, Zurich, Switzerland; 24 Center for Integrative Human Physiology Zurich, University of Zurich, Zurich, Switzerland

**Keywords:** Autism, Autism spectrum disorder, Social brain, fMRI, Mentalizing, Theory of mind, Animated shapes, Development, Multi-site

## Abstract

**Background:**

Autism spectrum disorder (ASD) is a neurodevelopmental condition with key deficits in social functioning. It is widely assumed that the biological underpinnings of social impairment are neurofunctional alterations in the “social brain,” a neural circuitry involved in inferring the mental state of a social partner. However, previous evidence comes from small-scale studies and findings have been mixed. We therefore carried out the to-date largest study on neural correlates of mentalizing in ASD.

**Methods:**

As part of the Longitudinal European Autism Project, we performed functional magnetic resonance imaging at six European sites in a large, well-powered, and deeply phenotyped sample of individuals with ASD (*N* = 205) and typically developing (TD) individuals (*N* = 189) aged 6 to 30 years. We presented an animated shapes task to assess and comprehensively characterize social brain activation during mentalizing. We tested for effects of age, diagnosis, and their association with symptom measures, including a continuous measure of autistic traits.

**Results:**

We observed robust effects of task. Within the ASD sample, autistic traits were moderately associated with functional activation in one of the key regions of the social brain, the dorsomedial prefrontal cortex. However, there were no significant effects of diagnosis on task performance and no effects of age and diagnosis on social brain responses. Besides a lack of mean group differences, our data provide no evidence for meaningful differences in the distribution of brain response measures. Extensive control analyses suggest that the lack of case-control differences was not due to a variety of potential confounders.

**Conclusions:**

Contrary to prior reports, this large-scale study does not support the assumption that altered social brain activation during mentalizing forms a common neural marker of ASD, at least with the paradigm we employed. Yet, autistic individuals show socio-behavioral deficits. Our work therefore highlights the need to interrogate social brain function with other brain measures, such as connectivity and network-based approaches, using other paradigms, or applying complementary analysis approaches to assess individual differences in this heterogeneous condition.

## Background

Atypicalities in social communication and interaction are a hallmark of autism spectrum disorder (ASD), a neurodevelopmental condition with onset in early childhood. The ability to recognize the mental state of a social partner, also referred to as theory of mind (ToM), has long been posited to be altered in autism in the “mind-blindness” account of ASD [1]. These deficits are suggested to be exacerbated during on-line mentalizing, e.g., during the instantaneous use of ToM in real-life social interactions, while impairments in explicit mentalizing, such as the instructed reasoning about mental states, can be related to (and compensated by) executive function abilities [2–6].

The mind-blindness account has stimulated a line of imaging research to explore the potential neurobiological underpinnings of mentalizing, and of social cognition in general, in ASD. In a seminal paper, Castelli and colleagues [7] presented short animations of geometric shapes whose movement patterns prompted mental state attribution (e.g., a triangle mocking another triangle [8]). In contrast to false-belief tasks, where the mental state of a social agent can be deduced by logical inference based on a given scenario, the animated shapes draw upon our irresistible tendency to attribute mental states—even to non-living beings devoid of facial or other human-like cues—in the very moment of watching [8, 9]. The authors found reduced activation in a sample of 10 adult ASD participants in regions of the so-called social brain, a neural network that is commonly activated in tasks involving mentalizing, especially the posterior superior temporal sulcus (pSTS), extending into the temporo-parietal junction (TPJ), and the dorsomedial prefrontal cortex (dmPFC [10]). This hypoactivation was paralleled by less accurate verbal descriptions of ToM animations. The authors suggested that mentalizing deficits in ASD might originate from a failure to adequately extract and process social meaning from sensory input. If validated in large samples, social brain responses to animated shapes could be exploited for biomarkers related to diagnosis, stratification, and/or treatment prognosis in ASD [11].

Numerous imaging studies on social cognition in ASD have since been performed. Here, the use of a wide variety of tasks and analysis procedures across different samples has resulted in a heterogeneous picture of neurofunctional alterations in ASD that are at times contradictory to the findings by Castelli et al. [7]. For instance, among those studies that employed an animated shapes task, methodological differences include (a) variations in ASD sample composition (e.g., 10 adults [mean age 33 ± 7.6 years, sex not specified] in [7]; 12 adolescents/adults [15 to 35 years, 2 females] in [12]; 13 adolescents [10 to 16 years, 2 females] in [13]; 17 adolescents/adults [13 to 23 years, 4 females] in [14]), (b) differences in imaging data analysis (e.g., different preprocessing routines with smoothing kernels ranging from 4.5 mm in [14] to 16 mm in [7], significance assessment on the whole-brain level [7, 12, 13] and/or within regions of interest [13, 14]), and even (c) differences in imaging modality (positron emission imaging in [7], fMRI in [12–14]). These and other studies on social cognition have produced mixed conclusions on ASD-related effects, ranging from reduced to excess activation in different brain regions within or outside the social brain, or no effect at all. In order to detect converging evidence, systematic meta-analyses have been performed but results vary with the studies included (e.g., [15–19]). Thus, while several candidate regions such as the medial prefrontal cortex, pSTS, amygdala, insula, fusiform face area, and inferior frontal gyrus (IFG) have been highlighted in the context of altered social information processing in ASD, the overall picture remains inconclusive.

An important source of variance in the ASD imaging literature is age, which needs to be addressed explicitly when studying developmental samples. The literature points to higher activation of frontal areas during adolescence compared to adulthood in typically developing individuals, which might reflect ongoing prefrontal maturation and less efficient inhibitory control in adolescence [20, 21]. However, this effect is not specific for social cognition and more research is warranted to address the effect of age on social brain function and its interaction with the autistic condition.

More recently, large-scale multicenter studies have been launched using more representative samples with higher statistical power. These have so far been limited to resting state imaging data [22, 23] which do not allow to draw conclusions on time-locked functional responses to external stimuli or task demands.

We have therefore extended previous large-scale efforts to include brain activation measures in the Longitudinal European Autism Project (LEAP [24]) where a well-powered, representative, and deeply phenotyped sample of participants with ASD and typically developing (TD) individuals has been characterized from childhood to adulthood. This unique cohort allows to address several key limitations of earlier studies, such as small sample size and low statistical power [25], restriction to specific age ranges, exclusion of the broader autism phenotype involving comorbidities, and limited clinical, psychological, and biological characterization for stratification analysis. In the current study, our aim was to discover and validate neurofunctional markers of social cognition alterations in ASD as a first step for biomarker discovery. The animated shapes task was chosen as one of four neurocognitive paradigms in this large study, given the promising findings in earlier reports that suggest high construct validity for on-line mentalizing deficits in ASD [7, 26, 27], and due to its good applicability across age ranges and intellectual abilities. Here, we used an adapted version of the task [27] that was recently shown to have reproducible effects on functional activation [28] and to be sensitive for autism-related traits [29]. Functional responses were comprehensively assessed as changes in brain activation and related to age and clinical status. We also undertook a dimensional analysis approach to investigate the influence of autism-related traits on social brain development. We expected individuals with ASD or with higher autism-related traits to show reduced regional activation in key areas of the social brain in response to the animated shapes [7, 13, 14]. Regarding the effect of age independent from diagnostic group, we expected younger participants to show higher frontal activation, reflecting a stronger involvement of areas implicated in executive control [20, 21].

## Methods

### Sample

Participants were part of EU-AIMS LEAP, a large multicenter European initiative aimed at the identification of biomarkers in ASD [24]. The study comprises 437 individuals with ASD and 300 TD individuals, both males and females, aged between 6 and 30 years. Participants underwent comprehensive clinical, cognitive, and MRI assessment at one of the following six centers: Institute of Psychiatry, Psychology and Neuroscience, King’s College London, UK; Autism Research Centre, University of Cambridge, UK; Radboud University Nijmegen Medical Centre, the Netherlands; University Medical Centre Utrecht, the Netherlands; Central Institute of Mental Health, Mannheim, Germany; and University Campus Bio-Medico of Rome, Italy. The study was approved by the local ethical committees of participating centers, and written informed consent was obtained from all participants or their legal guardians (for participants < 18 years). Individuals with ASD were included in the ASD sample based on an existing clinical diagnosis according to DSM-IV [30], DSM-IV-TR [31], DSM-5 [32], or ICD-10 [33]. Given the better accuracy of clinical judgments [34], individuals with ASD were not excluded if they did not reach the cutoff scores on the Autism Diagnostic Observation Schedule (ADOS [35]) or the Autism Diagnostic Interview-Revised (ADI-R [36]) during clinical characterization. For further details about inclusion and exclusion criteria and for a comprehensive clinical characterization of the LEAP cohort, we refer to Charman et al. [37]. For further details on the study design, we refer to Loth et al. [24]. In the present study, we selected all participants with an IQ > 75 for whom a structural and the task fMRI scan were available (*n*_TD_ = 231, *n*_ASD_ = 273). Participants with structural anomalies (*n* = 8), an incomplete task fMRI scan (*n* = 5), excessive head motion during the task fMRI scan (*n* = 74; defined as more than 20% of frames with a framewise displacement (FD) > 0.5 mm; Jenkinson et al. [38]), incomplete information (*n* = 11), and/or corrupted datasets due to technical failure (*n* = 16) were excluded. This resulted in the inclusion of 394 individuals, 205 individuals with ASD and 189 TD individuals, in our analyses (see Table 1 for an overview over key descriptive variables for the full sample, and Additional file 1: Table S1 for a sample description split by age group). Standard operation and quality control procedures are detailed in Additional file 1.

**Table 1:** Sample description

	ASD	TD	Statistics: ASD vs. TD
Total *n*	205	189	
Demographics			
Sex (male/female)	151/54	123/66	*χ*^2^(1) = 3.42, *p* = .079
Age (years)	17.9 ± 5.4 (7.1–30.6)	18.0 ± 5.5 (7.6–31.0)	*t*(392) = .32, *p* = .749
IQ (full IQ)	107.1 ± 14.2 (75.6–148.0)	108.5 ± 12.1 (76.8–142.0)	*t*(390) = 1.05, *p* = .295
Handedness (right/left/ambidext/unknown)	146/22/8/29	131/16/4/38	*χ*^2^(3) = 3.66, *p* = .301
Medication use (% of subjects)^1^	40.5	5.8	*χ*^2^(1) = 62.37, *p* < .001
In-scanner performance			
Mean framewise displacement (FD; in mm)^2^	.14 ± .08 (.03–.47)	.12 ± .08 (.03–.42)	*t*(392) = 1.88, *p* = .061
Volumes with FD > 0.5 mm (%)	3.55 ± 4.74 (0–19.40)	3.17 ± 4.33 (0–19.73)	*t*(392) = .82, *p* = .412
Signal-to-noise ratio	9.7 ± 1.3 (6.5–13.8)	9.9 ± 1.4 (6.5–13.8)	*t*(392) = 1.42, *p* = .155
Task accuracy	.81 ±.13 (.33–1.0)	.83 ± .13 (0–1.0)	*t*(392) = 1.30, *p* = .194
Clinical characteristics			
ADI-R^3^			
Social interaction	15.5 ± 6.7 (0–29)		
Communication	12.6 ± 5.6 (0–26)		
RRB	4.5 ± 2.6 (0–12)		
ADOS-2^4^			
Social affect	5.7 ± 2.5 (1–10)		
RRB	4.7 ± 2.6 (1–10)		
Total	5.0 ± 2.6 (1–10)		
SRS-2 (parent report)^5^			
Raw score	86.1 ± 30.6 (21–163)	19.9 ± 14.4 (1–74)	*t*(245) = 19.07, *p* < .001
*T* score	69.2 ± 12.1 (43–90)	45.1 ± 5.8 (37–66)	*t*(245) = 17.54, *p* < .001
DAWBA comorbidities^6^			
ADHD symptoms	1.6 ± 1.6 (0–5)	.2 ± .7 (0–3)	*t*(211) = 7.25, *p* < .001
Depression symptoms	1.1 ± 1.3 (0–5)	.4 ± .7 (0–4)	*t*(311) = 5.66, *p* < .001
Anxiety symptoms	2.5 ± 1.3 (0–5)	1.2 ± .9 (0–4)	*t*(348) = 11.06, *p* < .001

Participant characteristics, split by sample and age group. If not otherwise indicated, numbers reflect mean ± standard deviation, followed by value range (minimum–maximum)

^1^Percentage of participants taking medication prescribed for behavioral or neurological problems. Medication data was available for 132 ASD and 78 TD participants. See Additional file 1: Table S1 more information on medication

^2^Motion was assessed as mean framewise displacement according to [38]

^3^Autism Diagnostic Interview-Revised (ADI-R [36]) scores were computed for reciprocal interaction (social interaction), communication, and restrictive, repetitive stereotyped behaviors and interests (RRB). ADI-R scores reflect historical symptom severity (age 4–5 years) and were available for 197 ASD participants

^4^Autism Diagnostic Observation Schedule 2 (ADOS-2 [35]). Calibrated severity scores [39] were computed for social affect, RRB, and the overall total score. ADOS-2 scores reflect current symptom severity and were available for 198 ASD participants

^5^Total raw and total *T* score (sex and age normalized) on the Social Responsiveness Scale-2 (SRS-2 [40]). SRS-2 scores were available for 247 participants (i.e., TD adults are excluded from this measure; see Additional file 1 for analyses using self-reported SRS-2 scores). The raw SRS-2 scores were used in our analyses

^6^Comorbid symptoms of ADHD, depression, and anxiety were assessed with the Development and Well-Being Assessment (DAWBA [41]), generating six levels (ordinal scores 0 to 5) of prediction of the probability of a disorder (~ 0.1%, ~ 0.5%, ~ 3%, ~ 15%, ~ 50%, > 70%). DAWBA scores were available for 167 ASD and 146 TD participants for depression, 140 ASD and 73 TD participants for ADHD, and 183 ASD and 167 TD participants for anxiety

### Power analysis

We performed a power analysis for our sample using GPower [42] to assess statistical sensitivity to detect an effect of group (TD vs. ASD) or an effect of age in a single hypothesis test (e.g., single voxel or region of interest) at a type I error rate of *α* = .05 while accounting for covariates of no interest (sex and site, see below; statistical test in GPower: linear multiple regression, fixed model, *R*^2^ increase; number of tested predictors, 1; total number of predictors, 8). Based on this model, the study has a power of 80% to detect a standardized effect size of *f*^2^ ≥ .02 and a power of 95% to detect a standardized effect size of *f*^2^ ≥ .03, with *f*^2^ ≥ .02 denoting a small effect and *f*^2^ ≥ .15 denoting a medium effect [43]. In addition, with our large sample of *N* > 400, this study is less prone to false positives and overestimation of effect sizes, in particular in the scenario of weak diffuse effects which is typical for task fMRI [44].

### Clinical measures

Participants in the ASD group had an existing clinical diagnosis of ASD according to the DSM-IV-TR/ICD-10 or DSM-5 criteria. ASD symptoms were comprehensively assessed using the Autism Diagnostic Interview-Revised (ADI-R [36]) and Autism Diagnostic Observation Schedule 2 (ADOS-2 [35]). In the current sample, 85% of ASD individuals met the diagnostic threshold on the ADI-R or ADOS-2, while 49 % met the cutoff on both instruments. Individuals who did not reach the cutoff on either scale were included on the basis of careful clinical judgment made by expert clinicians in the participating ASD specialist centers. We used the parent-reported total raw score on the Social Responsiveness Scale Second Edition (SRS-2 [40]) as a continuous measure for autism traits across all participants. The SRS-2 allows for the assessment of autism traits across clinical and non-clinical samples and includes 65 questions about autistic behaviors, generating scores ranging from 0 to 195, with higher scores indicating the presence of pronounced traits. This measure was available for all ASD and TD individuals except for TD adults where only the self-report was assessed. Analyses on SRS-2 scores including TD adults are presented in Additional file 1.

### Task

We assessed functional brain responses during an adapted version of the animated shapes task [27, 29] used in Castelli et al. [7] which was presented as part of a structural and functional imaging battery [24]. The task consisted of short video clips (26 to 48 s) featuring two triangles whose movement patterns reflected increasing levels of mental state attribution according to three conditions: (1) no or little mental state attribution for random movement (e.g., floating around), (2) perception of agency and hence of mental states for goal-directed movement (e.g., chasing), and (3) perception of complex mental states involving theory of mind (e.g., cheating). Four videos per condition were presented in a pseudorandomized order, with no more than two animations of the same condition presented in a row. Participants passively watched each video clip. In the subsequent response phase (5 s), participants were asked to categorize the animation to one of the three conditions by selecting the corresponding icon by button press. Selected icons were highlighted by a red frame for the duration of 1 s, followed by a fixation cross and a variable jitter (M = 996 ms, SD = 418 ms). No feedback on categorization accuracy was given. The prompted categorization during fMRI deviated from the procedure in Castelli et al. where subjects gave verbal descriptions, i.e., narratives, after each animation. The categorization used in the current study has previously been shown to be a sensitive and objective test of on-line mentalizing with the advantage of a faster assessment and more objective analysis of the participant’s response [27]. Participants were familiarized to the task in a standardized training session before scanning, including three practice video clips. Task performance during the fMRI scan was evaluated in terms of overall categorization accuracy and accuracy of ToM video categorization (see Additional file 1 for additional age group-specific analyses). Analyses were performed in SPSS (IBM, version 22) using univariate analyses of covariance to assess the effects of diagnosis and age while controlling for sex, site, and IQ. See Additional file 1 for more details on stimuli, trial structure, and task instruction.

### MRI data acquisition

Data were acquired on 3-T MRI scanners, and acquisition protocols were harmonized across sites as closely as possible. Functional data was collected using an echo-planar imaging (EPI) sequence, and structural images were acquired with a high-resolution T1-weighted magnetization-prepared rapid gradient echo sequence. Data were subjected to an extensive quality assessment pipeline. Detailed information on scanning parameters and data quality control procedures is given in Additional file 1.

### fMRI data analysis

#### Preprocessing

Functional imaging data were preprocessed using standard analysis routines implemented in SPM12 (http://www.fil.ion.ucl.ac.uk/spm/), including slice-time correction, a two-step realignment procedure, unified segmentation and normalization to standard stereotactic space as defined by the Montreal Neurological Institute (MNI), and smoothing with an 8-mm full-width-at-half-maximum Gaussian Kernel.

#### Subject-level activation analysis

Task conditions were modeled as boxcar functions that accounted for the presentation of videos and its parametric modulation. Increasing values of the parametric modulator (i.e., 1, 2, and 3) coded for increasing mentalizing demands in random, goal-directed, and ToM conditions, respectively. This approach deviates from previous ASD studies where the ToM condition was compared to the low-level baseline condition (i.e., random movement) while ignoring the goal-directed condition [7, 12, 13, 28, 29, 45, 46]. The current approach comes with the advantage that it draws on all available data, thereby maximizing statistical power and aligning with the assumption of a gradual increase of mentalizing demands across conditions [26]. We additionally modeled task effects of no interest, i.e., response selection and execution during video categorization, with boxcar functions of variable durations depending on the participant’s response time. Task regressors were convolved with the canonical hemodynamic response function (HRF) and subjected as predictors to a general linear model (GLM), along with six realignment parameters to account for head motion. During first-level model estimation, data was high-pass filtered with a cutoff of 256 s, and an autoregressive model of the first order was applied. To identify brain responses reflecting sensitivity to social significance, the effect of the parametric modulator was contrasted to the implicit baseline.

We additionally tested for case-control differences in brain activation obtained from the original approach where animations were modeled as boxcar functions and assigned to three condition-specific regressors of interest (random, goal-directed, ToM). All remaining steps of model specification and estimation were identical. Individual contrast images were calculated for the contrast ToM > random and ToM > goal-directed, respectively.

#### Group-level statistical inference

Resulting contrast images were subjected to a GLM to assess the within-subject effect of task and the between-subject effects of diagnosis and age while controlling for effects of sex and site. Age-by-diagnosis interactions were tested in an additional GLM. To assess the effect of autism traits, SRS-2 raw scores were added as an additional covariate in a separate model. Note that group was accounted for in this model, which ensures that effects were not driven by a mere difference in group means. In order to account for between-subject effects that specifically occur during development or only emerge in adulthood, two subsamples were defined: a *youth sample* (< 18 years) and an *adult sample* (≥ 18 years). All analyses were repeated separately in both subsamples. Across analyses, effects were evaluated at a statistical threshold of *p* = 0.05, family-wise error corrected (FWE) on a voxel level across the whole brain and within the combined mask of the right pSTS and dmPFC using small volume correction (SVC; 503 voxels). Case-control analyses were complemented by tests for equality of distribution at selected peak voxels, using the Kolmogorov-Smirnov test with a significance level of *α* = .05 implemented as the *ks2stat* function in Matlab (version R2018b, The MathWorks, Inc.).

#### Control analyses

We performed control analyses to investigate whether the results were affected by various potentially influencing variables, such as demographic (acquisition site, IQ) and clinical characteristics (medication, comorbidity, current diagnostic status), task performance (categorization accuracy), functional image quality (motion and signal-to-noise ratio), and SRS informant (SRS self-report, available for adults in the ASD groups and for adolescents and adults in the TD group). We also explored whether ASD-related effects were modulated by sex (i.e., sex by diagnosis interaction). Finally, we tested whether functional brain activation related to the participants’ categorization performance. See Additional file 1 for details on all control analyses.

### Narrative performance

Following Castelli and colleagues [7], we assessed verbal descriptions of the animations as an additional behavioral measure. These narratives might be particularly sensitive to spontaneous mentalizing since participants are not explicitly prompted to categorize the animation. Narratives were assessed for the same animations in a separate cognitive test session that preceded the fMRI scanning on a separate day. In order to minimize participant burden, 10 animations were used (4× ToM, 4× GD, 2× random). In short, participants were asked to spontaneously describe what was happening in each animation while watching. The responses were audio typed and analyzed based on a standardized scoring system (see Additional file 1). Here, we use the participant’s mentalizing score which reflects the use of mental state terms (score of 0: absence of any mental state term, score of 1: terms that denote intentionality, e.g., “the triangle chases the other triangle,” score of 2: terms that denote interactions involving theory of mind, e.g., “the small triangle tries to convince the big triangle to go out”). The mentalizing score therefore parallels the three task conditions and can thus be compared to the categorization performance during fMRI scanning.

## Results

### Behavioral performance

**Fig. 1:**
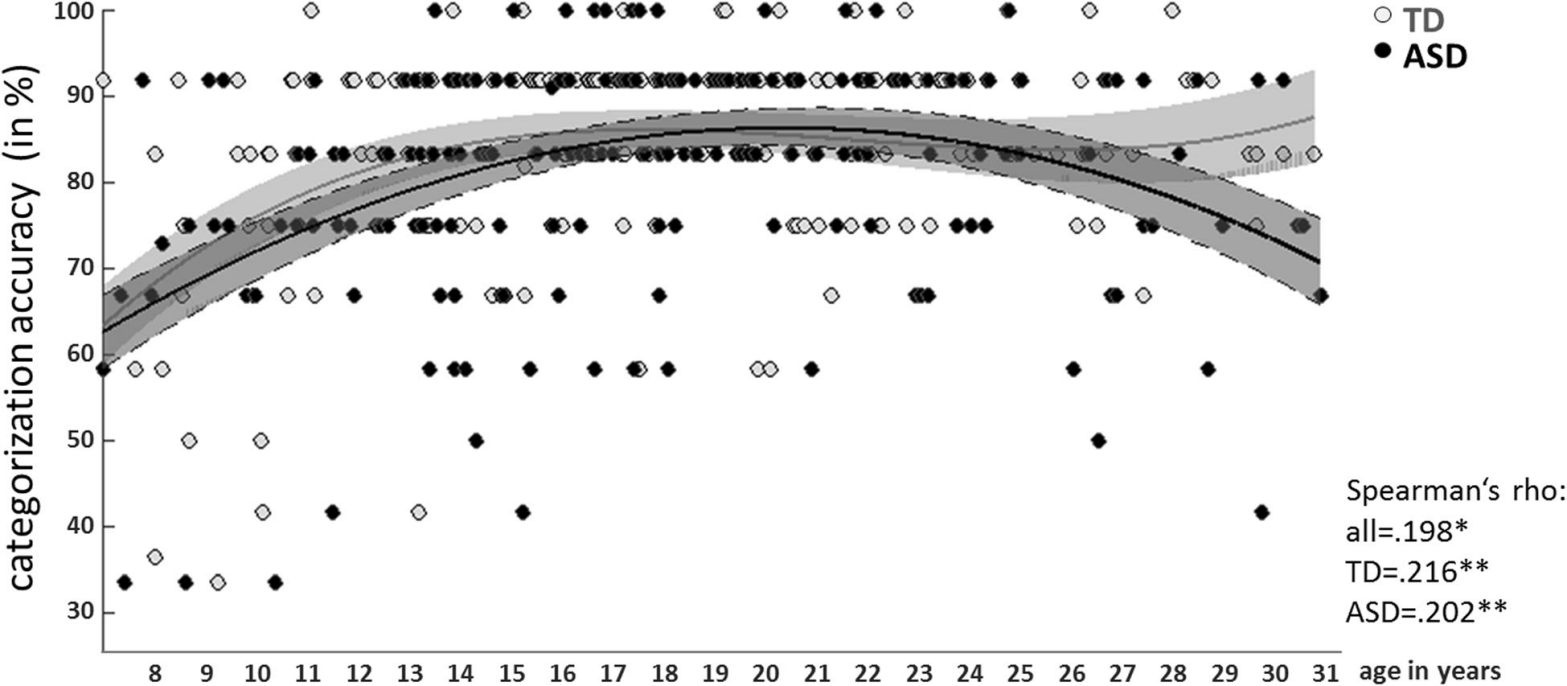
Overall categorization accuracy. Linear least square regression fits (third level polynomial) for TD (gray) and ASD (black) are shown for the full sample. Shaded areas represent 95% confidence intervals. Non-parametric correlation coefficients (Spearman’s rho) are reported for all subjects and separately for TD and ASD subjects.*p < .05, **p < .01

Statistical information on categorization performance during fMRI scanning is detailed in Table 2. Diagnostic groups did not differ in overall categorization accuracy or ToM video categorization accuracy. In contrast, we observed an effect of age, with better performance in older subjects. Due to the skewed distribution of values, non-parametric correlations with age are reported in Fig. 1.

**Table 2:** Video categorization accuracy for the full sample, youth sample (< 18 years of age), and adult sample (≥ 18 years of age)

Accuracy (in %)	ASD	TD	ME diagnosis	ME age	IA age × diagnosis
Overall	81.4 ± 13.4	83.2 ± 13.4	*F*_(1,381)_ = .383, *p* = .536	***F***_**(1,381)**_**= 20.220,*p*< .001**	*F*_(1,381)_ = .727, *p* = .394
ToM video	82.7 ± 20.5	86.2 ± 19.0	*F*_(1,381)_ = 2.181, *p* = .141	***F***_**(1,381)**_**= 28.377,*p*< .001**	*F*_(1,381)_ = .097, *p* = .755

Significant effects printed in bold; *ASD* autism spectrum disorder, *TD* typical development, *ME* main effect, *IA* interaction effect

Similarly to categorization performance, behavioral performance on the narratives (i.e., mean mentalizing scores for the three conditions) did not differ between cases and controls, irrespective of age (*F*_(1,338)_ ≤ 1.00, *p* ≥ .318). We performed a mixed multilevel analysis to test the association between fMRI categorization and mentalizing ratings along with the influence of diagnostic status and age while controlling for sex and site. Results suggest a significant association between categorization and mentalizing scores (*t* = 20.798, *p* < .001), with no effect of age (*t* = 1.632, *p* = .103) or diagnosis (*t* = .71, *p* = .478). We additionally tested how well mean categorization accuracy corresponded to mean mentalizing scores for ToM animations. Due to the low range of possible values, non-parametric correlation analysis was used, which suggested a significant association (Kendall’s tau = .145, *p* = .001; Spearman’s rho = .171, *p* = .001).

### Brain activation

**Fig. 2:**
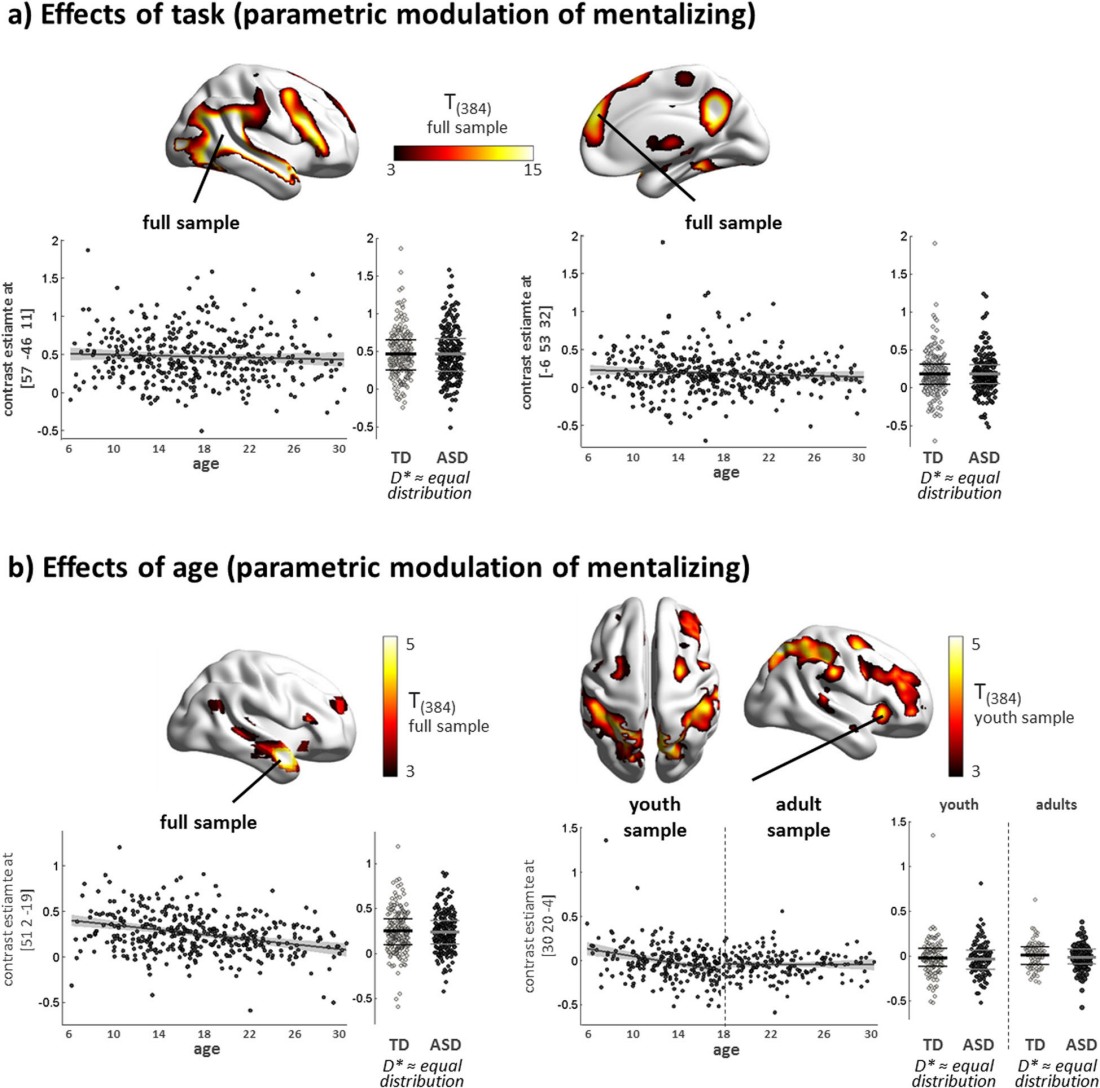
Functional activation to spontaneous mentalizing elicited by increasing social significance of animated video clips. **a** Render brains illustrate the positive effect of task (i.e., effect of increasing social significance) in the full sample. Scatter plots highlight functional responses in selected peak voxels in the right pSTS (left) and dmPFC (right). **b** Render brains illustrate the positive effect of age in the full sample (left) and youth sample (right), complemented by scatter plots of peak voxels in the right anterior temporal pole (left) and right anterior insula (right). Associations with age are displayed using a linear model fit (95% confidence interval indicated as shaded area). Distributions of peak voxel activation in cases and controls were compared using the Kolmogorov-Smirnov test, which suggested no evidence for unequal distributions (statistics for peak at [57, − 46, 11], full sample: *D*_(205,189)_* = .065, *p* = .789; for peak at [− 6, 53, 32], full sample: *D*_(205,189)_* = .044, *p* = .990, for peak at [51, 2, − 19], full sample: *D*_(205,189)_* = .061, *p* = .853; for peak at [30, 20, − 4], youth sample: *D*_(111,105)_* = .081, *p* = .855; adult sample: *D*_(93,84)_* = .140, *p* = .327). Distribution mean and 25th/75th percentiles are indicated as thick and thin lines within bee swarm plots, respectively. For illustration purposes, render brains are displayed at a significance threshold of *t* = 3 using BrainNet Viewer (http://www.nitrc.org/projects/bnv/). TD, typically developing; ASD, autism spectrum disorder; D*, test statistic of the Kolmogorov-Smirnov test

Increasing mentalizing demands across conditions led to robust activation of key regions of the social brain, including pSTS and dmPFC, as predicted (Fig. 2a, Table 3, Additional file 1: Figure S3 and S4). We observed distinct effects of age in the full and youth samples (Fig. 2b). While activation in the right anterior temporal sulcus and temporal pole decreased across the full age range (6–30 years), a specific decrease of activation was detected in the youth sample (6–18 years) in regions typically involved in the dorsal and ventral attention networks (e.g., frontal eye fields, intraparietal sulcus, anterior insula).

In the categorical analyses, brain responses to increasing mentalizing demands did not differ between diagnostic groups on the whole-brain level and within ROIs across age groups (ROI statistics for the full sample are as follows: right pSTS, main effect of diagnostic group: *F* ≤ 3.11, *p*_FWE_ (SVC) ≥ .949, diagnostic group × age interaction: *F* ≤ 6.35, *p*_FWE_ (SVC) ≥ .520; dmPFC, main effect of diagnostic group: *F* ≤ 4.34, *p*_FWE_ (SVC) ≥ .828; diagnostic group × age interaction: *F* ≤ 7.33, *p*_FWE_ (SVC) > .379). Cases and controls did not differ in the distribution of functional responses in selected peak voxels (see Fig. 2; all *p* > .05), and visual inspection of distributions did not suggest the formation of meaningful subgroups.

**Fig. 3:**
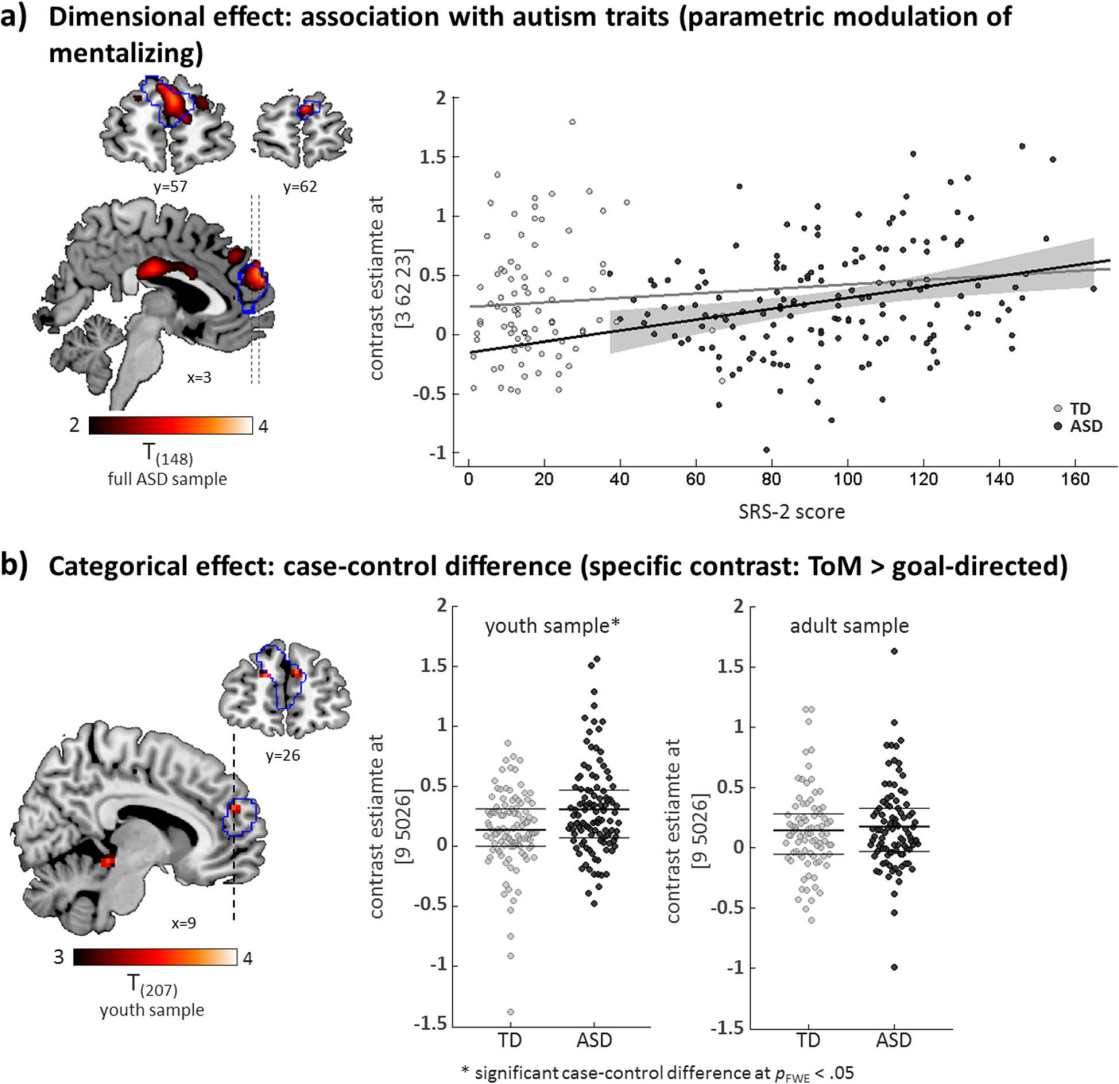
**a** Brain sections (left) illustrate the association of a continuous measure of autism traits, as assessed as parent-reported scores of the Social Responsiveness Scale-2 (SRS-2), with functional responses to increasing mentalizing demands in the dmPFC in individuals with ASD. The outline of the ROI in the dmPFC is displayed in blue. A scatter plot (right) demonstrates the distribution of autism trait scores and peak voxel activation in cases and controls, including linear model fits for each group. The 95% confidence interval for the significant linear model fit in the ASD group is displayed in gray. **b** Brain sections (left) illustrate the case-control difference for the specific contrast ToM > goal-directed in the dmPFC ROI (outlined in blue) in the youth sample. Scatter plots (right) display the distributions of peak voxel contrast estimates in the youth and adult sample, with mean and inner quartiles (25th to 75th percentile) indicated as thick and thin lines, respectively

In contrast, the dimensional analysis uncovered an increase in dmPFC responses with increasing autism traits (full sample: peak voxel at *x* = 3, *y* = 62, *z* = 23; *t* = 3.88, *p*_FWE_ (SVC) = .011). This effect was driven by the ASD group (statistical analysis on peak voxel estimate in ASD group: *F*_(1,150)_ = 14.53, *p* < .001; in TD group: *F*_(1,77)_ = .04, *p* = .841; Fig. 3a). See Table 3 for a detailed list of brain regions, coordinates, and statistics.

**Table 3:** Whole-brain parametric effects of increasing mentalizing demands on brain activation

Effect/contrast	Sample	*k*	Region	*x*	*y*	*z*	*t*	*p*_corr_
Within-subject effects								
Effect of task								
Mentalizing demands: parametric increase	Full	3036	Superior temporal gyrus	57	− 46	11	28.69	< .001
			Middle occipital gyrus [area hOc4lp]	27	− 91	2	24.99	< .001
			Superior temporal gyrus	48	− 22	− 7	22.04	< .001
			Inferior temporal gyrus [area FG4]	42	− 52	− 13	21.25	< .001
			Temporal pole	54	14	− 19	16.71	< .001
		4251	Middle temporal gyrus	− 54	− 52	14	26.84	< .001
			Middle occipital gyrus [area hOc4lp]	− 27	− 91	− 4	23.03	< .001
			Fusiform gyrus [area FG4]	− 39	− 55	− 10	21.55	< .001
			Cerebellum [lobule VIIa crusI (hem)]	− 24	− 76	− 34	16.49	< .001
			Inferior frontal gyrus, pars triangularis	− 51	20	20	16.40	< .001
		1115	Precuneus	6	− 52	38	18.85	< .001
		868	Inferior frontal gyrus, pars triangularis [area 45]	51	26	17	14.96	< .001
			Precentral gyrus	45	5	50	12.57	< .001
		925	Dorsomedial prefrontal gyrus	− 6	53	32	13.98	< .001
			Supplementary motor area	9	17	65	10.82	< .001
		56	Cerebellum [lobule IX (hem)]	− 6	− 52	− 40	11.02	< .001
		553	Thalamus [thalamus: prefrontal]	9	− 13	8	9.46	< .001
		27	Cerebellum vermis [lobule I IV (hem)]	0	− 46	− 16	6.06	< .001
		6	Middle cingulate cortex	0	− 16	41	5.44	.001
		2	Middle frontal gyrus	− 24	23	44	4.60	.025
Between-subject effects								
Effect of age								
Age: linear decrease	Full	12	Medial temporal pole	51	2	− 19	5.04	.004
Age: linear decrease	Youth	249	Superior parietal lobule [area 7P (SPL)]	− 27	− 58	56	6.37	.000
		207	Superior parietal lobule [area 7P (SPL)]	18	− 67	53	5.75	.000
			Precuneus	− 6	− 76	44	5.45	.001
			Superior parietal lobule [area 7A (SPL)]	18	− 58	62	5.30	.002
		35	Inferior parietal lobule [area hIP1 (IPS)]	− 36	− 40	38	5.14	.004
			Inferior parietal lobule [area PFt (IPL)]	− 48	− 34	41	4.78	.017
		35	Inferior parietal lobule [area 2]	48	− 34	47	5.01	.007
		13	Middle cingulate gyrus	6	26	38	4.85	.013
			Anterior cingulate gyrus	6	32	26	4.60	.034
		20	Superior frontal gyrus	24	− 1	47	4.83	.014
		11	Anterior insula	30	20	− 4	4.77	.017
		5	Supramarginal gyrus (area PFt (IPL)]	63	− 22	35	4.72	.021
		8	Inferior parietal lobule [area 7PC (SPL)]	33	− 46	53	4.72	.021
		2	Middle frontal gyrus	45	38	20	4.60	.034
		1	Postcentral gyrus [area 4p]	33	− 31	50	4.57	.037
Effect of diagnosis (categorical)								
	No sign. effect							
Effect of parent-reported autism traits (dimensional)								
Linear increase	Full (no TD adult^1^)	4	Inferior parietal lobule [area PFm (IPL)]	54	− 55	38	4.82	.021
		10_SVC_	dmPFC (combined mask)	3	62	23	3.88	.011_SVC_
		15_SVC_	dmPFC (combined mask)	− 9	53	38	3.49	.035_SVC_
Linear increase	Youth	23_SVC_	dmPFC (combined mask)	3	56	26	3.77	.016_SVC_
		8_SVC_	dmPFC (combined mask)	− 15	50	35	3.43	.046_SVC_

Regions were classified according to the Automated Anatomical Labeling Atlas [47]. If applicable, functional labels were added in square brackets based on Anatomical Probability Maps (Anatomy toolbox [48]). *x*-, *y*-, and *z*-coordinates (MNI) and statistical information refer to peak voxels in the identified clusters. *p* values are adjusted for family-wise error correction for multiple comparisons across the whole brain, or across the combined mask of the right pSTS and dmPFC using small volume correction (SVC). Age and sex were included as covariates in the analysis. *SPL* superior parietal lobule, *IPS* intraparietal sulcus, *IPL* inferior parietal lobule, *TD* typically developing, *pSTS* posterior superior temporal sulcus, *dmPFC* dorsomedial prefrontal cortex

^1^ no parent-reported SRS-scores available for TD adults

Our additional analyses involving the ToM > random contrast did not yield any case-control difference on the whole-brain level and within regions of interest across samples (all *p*_FWE_ > .277). The contrast involving the high-level baseline condition (ToM > goal-directed) revealed a small cluster within the dmPFC in the youth sample where individuals with ASD showed a stronger response compared with TD individuals (peak voxel at *x* = 9, *y* = 50, *z* = 26; *F* = 13.56, *p*_FWE_ (SVC) = .034; Fig. 3b). No other cluster emerged across search masks and samples (all *p*_FWE_ > .251). Dimensional analyses using these contrasts replicated the association within the dmPFC observed in the parametric modulation approach (ToM > random, full sample: peak voxel at *x* = 3, *y* = 62, *z* = 23; *t* = 3.35, *p*_FWE_ (SVC) = .057; youth sample: peak voxel at *x* = 15, *y* = 50, *z* = 35; *t* = 4.20, *p*_FWE_ (SVC) = .004). See Additional file 1: Table S4 for a full list of categorical and dimensional effects for the specific contrasts.

### Control analyses

The between-subject effects of age and diagnosis reported above were largely robust to the inclusion of additional control variables. As a notable exception, we observed an influence of site on the association between dmPFC and autism traits; the association dropped below the significance threshold when skipping one of the major contributing sites (KCL). The direction of the association, however, did not change (Additional file 1: Figure S5). Follow-up analyses suggest that the KCL site effect is most plausibly explained by the fact that the KCL site contributed a (relatively) higher number of (relatively) more severely affected individuals with ASD, which boosted the association between dmPFC activation and autism trait scores. Regarding effects of medication, the exclusion of medicated individuals resulted in an attenuation of the dimensional effect in the dmPFC, which however could not be attributed to symptom severity. The direct comparison of medicated and unmedicated individuals with ASD revealed a diminished response of the insula and inferior frontal cortex in medicated children and adolescents (Additional file 1: Figure S6). No effects were observed within the canonical social brain network. We also did not observe a modulation of ASD-related effects by sex. Likewise, follow-up analyses do not suggest an impact of categorization accuracy on the association between dmPFC and autism traits. See Additional file 1 for detailed information on the results of all control analyses.

## Discussion

In this to date largest study on the neurofunctional development of the social brain, we characterized social brain activation during mentalizing in a deeply phenotyped sample of individuals with ASD and TD controls. Besides showing a robust effect of task, we demonstrate (1) that functional responses of core regions of the social brain are well developed by the age of 6 while age-related changes occur in a distributed set of brain regions typically implicated in attention and executive control, (2) that categorical case-control comparisons between TD and ASD participants do not reveal clear-cut group differences in mean and distribution of functional activation measures, and (3) that a dimensional analysis approach might offer higher sensitivity to detect ASD-related effects.

### Robust effects of social animations on functional activation

Across both groups, we replicated the effect of increased activation in key regions of the social brain [7, 10] that scaled with the mentalizing demands imposed by the animations. Resulting activation patterns are highly similar to those obtained from the comparison of the ToM condition to the low-level baseline condition, which suggests that effects are mainly driven by the ToM condition. Activation was strongest in the bilateral pSTS and adjacent temporal and occipital cortices, reflecting the central role of sensory bottom-up processing in this task where social meaning is extracted from the spatial constellation of the moving shapes. We also observed robust responses of the dmPFC, IFG, precuneus, and temporal poles, as previously described [10, 29].

### Effects of age

Across both groups, younger participants were less accurate in video categorization. However, this age effect in behavior was not reflected on the level of the social brain circuitry where functional activation was largely unaffected by age. This is in contrast to several studies on mentalizing that reported higher social brain activation in younger subjects in various regions, such as medial frontal, inferior frontal, or temporal areas (e.g., [46, 49–51]). Common interpretations are less efficient processing or different cognitive strategies [52] that might parallel ongoing structural maturation [53]. In our large sample, however, these effects did not replicate, neither across the full sample nor when splitting the sample into two subsamples to approximate non-linear age effects, and despite the fact that we had a 95% power to detect small-to-moderate effects. An exception is the right temporal pole that showed a gradual decrease of activation from childhood to adulthood, potentially reflecting its protracted structural development [53]. We propose two explanations for the lack of age effects on social brain activation. First, while previous reports were fairly consistent in reporting stronger responses in younger individuals, they were less consistent in the localization of these effects. For instance, medial prefrontal effects ranged from subgenual to dorsal areas. This heterogeneity likely results from methodological differences and limited statistical power, along with selection and reporting biases (e.g., [25, 44, 54–57]), which questions the generalizability of previous findings [25]. Second, spontaneous mentalizing is a fundamentally basic skill akin to tracking beliefs, an ability already seen in toddlers ([58, 59], but see [60] for a critical discussion). This suggests an early development of its neurofunctional basis. In fact, a recent study demonstrated the social brain network to be functionally distinct and robustly recruited in 3-year-old children, independent from explicit mentalizing skills [61]. Similarly, a recent study in 50 children and adolescents did not find an effect of age on several measures of functional connectivity of the social brain [62]. Overall, these and our findings suggest that across study populations, the core neurofunctional network for mentalizing is largely set by the age of 6 and does not undergo gross changes from mid-childhood to adulthood. This does not preclude the existence of specific developmental alterations in conditions like ASD, as discussed below.

Outside the social brain, however, we did observe an effect of age. A distributed set of brain regions including the frontal eye fields, intraparietal sulcus, and anterior insula demonstrated a gradual decrease of activation from childhood to early adulthood. These regions have been identified as key hubs of the dorsal and ventral attention systems that support top-down controlled orienting of attention and bottom-up-mediated reallocation of attention to salient events [63]. Age effects were also evident in dorsolateral prefrontal areas that have been suggested to support the flexible switch between both attention systems [63]. According to our data, children and young adolescents might recruit attentional resources more strongly than older adolescents and adults when inferring social meaning from the animated shapes. Along with our observation of better task performance in adults, these findings suggest an important contribution of domain-general networks to social cognition [64].

### Effects of diagnosis

Contrary to our hypothesis, we did not observe robust case-control differences in both behavioral measures of mentalizing (i.e., categorization and narratives) and in social brain responses to increasing levels of mental state attribution. We also did not see a case-control difference when comparing the ToM condition to the low-level baseline condition. However, when comparing the ToM condition to the goal-directed condition, one cluster emerged within our dmPFC key region, showing a higher average response in ASD, which was restricted to the youth sample. Across these analyses, we did not see major differences in the distributions of functional responses between cases and controls or any indication for the formation of subgroups within the ASD sample. Beyond the categorical approach, we observed a moderate and positive association between a continuous measure of autistic traits and mentalizing-related activation in the dmPFC. This association was only observed in the ASD group and predicted higher brain responses in individuals with higher autistic traits, irrespective of age.

The lack of robust case-control differences needs to be discussed in the light of the positive findings by Castelli et al. [7] and others using the animated shapes task [12–14]. First, as discussed above in the context of non-replicable age effects, one possible explanation is that previously reported case-control differences in studies with low statistical power reflect idiosyncratic characteristics of the specific sample and experimental procedure. A recent study using simulated and empirical fMRI data provided compelling evidence that between-subject effects, such as case-control differences and brain-behavior relationships, are usually weak and spatially distributed [44]. It is plausible that ASD-related effects generally also follow this weak and diffuse pattern. As a result, commonly used sample sizes in earlier ASD studies (*n* = 10–30), which were sufficiently powered to detect strong and localized within-subject effects (e.g., effect of an experimental condition), might have lacked the statistical power to detect robust effects related to ASD. A consequence of this power failure is an overestimation of effect sizes, including spurious effects due to random error, which produced a heterogeneous pattern of putative ASD-related effects that do not replicate in follow-up studies. Second, the continuous revision of diagnostic criteria and the fact that the autism diagnosis is not stationary over time might further impact the comparability to older studies [65]. A third explanation is that earlier findings obtained in smaller, more homogeneous, and potentially more severely affected samples might relate to specific subsamples, or “strata”, within the ASD population. The LEAP cohort was purposely sampled to enable in-depth experimental characterization of potential biomarkers (including MRI scans) and therefore excluded individuals with low IQ (< 50) and most likely individuals with the most severe autistic presentations. However, the sample does allow us to investigate the heterogeneity of the ASD population with respect to age, sex, comorbidities, and symptom profiles. In the current study, we have not leveraged this heterogeneity for stratification purposes yet, but tested for differences in average response while controlling for these sources of variance, which might have resulted in reduced sensitivity for subsample-specific effects. For instance, despite covering the full range of symptom severity, the LEAP cohort is, on average, rather mildly affected, which might have contributed to the negative findings in case-control comparisons. However, our control analysis restricted to more severely affected individuals (see section 6.8 in the Additional file 1) did not change the results, which is not consistent with this potential explanation for our negative findings, at least in our sample. Fourth, despite extensive standard operation procedures, the multi-site acquisition design is a potential source of unwanted variance which might have similarly resulted in reduced sensitivity for smaller-sized effects [66]. We addressed this variance in our analyses which did not suggest a systematic confounding effect of site. Fifth, in contrast to Castelli et al. [7], participants were prompted to focus on the category of the animation, which introduces an explicit component to this otherwise implicit, or “spontaneous,” mentalizing task. It has been suggested that spontaneous mentalizing is the key difference between autism and typical development and that individuals with ASD can explicitly mentalize when prompted to do so [2]. The explicit categorization might therefore have reduced, or even abolished, mentalizing-related case-control differences in our study [67]. While this interpretation needs to be tested in future large-scale studies, it is challenged by earlier reports of behavioral case-control differences using the prompted categorization approach [13, 27]. In addition, our analyses suggest a significant association between prompted categorization and a measure of spontaneous mentalizing in the same individuals. Finally, the intriguingly simplistic yet powerful effect of the animated shapes is hypothesized to originate from an efficient, early developing mentalizing system that offers little room for manipulation by explicit strategies [9, 58], which is supported by neurofunctional evidence in TD participants [45].

Our categorical approach of comparing cases and controls therefore does not lend support to the hypothesis that social brain function during mentalizing elicited by animated shapes is a one-to-one correlate of social impairments in individuals with ASD. That said, however, both categorical and dimensional analyses converged on two small-to-moderate effects in our dmPFC key region. The mentalizing-specific activation in the dmPFC was stronger in younger individuals with ASD compared to TD individuals. Similarly, dmPFC activation increased with the degree to which an individual with ASD demonstrates autistic behavior as assessed by parent-reported autistic traits. While counterintuitive at first sight, a possible explanation is a greater need for a compensatory recruitment of the dmPFC in younger individuals and with increasing autism traits, an effect that was recently introduced as “camouflaging” in the context of mentalizing [68]. This interpretation is also in line with the absence of a dimensional effect in TD individuals, although this seems to be primarily due to the limited variance in autism trait scores. Supplemental analyses do not suggest an association of dmPFC responses with categorization accuracy, which can similarly be reconciled with the hypothesis of successful camouflaging. We acknowledge, however, that caution is warranted when interpreting our dimensional finding since our control analyses point to a potential effect of site and since this association occurs within the neurotypical range, as suggested by the lack of an overall case-control difference. Further studies are needed to follow up on this finding and its potential biological meaning.

What does our observation of largely overlapping social brain responses in individuals with ASD and TD individuals add to current theories of autism? The animated shapes task is hypothesized to mainly draw upon implicit mentalizing which is required for fast-paced real-life interactions [9, 58] and which is suggested to be specifically impaired in autistic individuals [2]. With the caveat of an explicit component introduced by the prompted categorization, our results provide no evidence of an altered recruitment of its neural correlates in autism, at least in terms of a consistent alteration in the strength of activation. This leaves open the possibility of idiosyncratic, non-converging alterations in brain activation which is not captured by conventional fMRI analyses based on group means [65, 69]. Likewise, the dysconnectivity account of autism postulates alterations in functional connectivity to underlie behavioral and clinical impairments [70]. Measures of functional connectivity and their modulation by mentalizing demands might therefore be more promising for biomarker research. Additional, likely multiple, causes might contribute to the exacerbation of socio-communicative impairments of autistic individuals in everyday life. These may include cascading effects of impairments in other domains (e.g., sensory abnormalities [71]), a lower propensity to adopt the intentional stance [72], reduced motivational salience of social and non-social stimuli [73], or direct or indirect effects of comorbid conditions (e.g., alexithymia [74]).

### Limitations

Despite an extensive set of control analyses, we cannot rule out additional sources of variance that remain unaddressed or poorly addressed, such as specific effects of medication, and might thereby prevent the detection of effects of interest despite our well-powered sample. Regarding sample characteristics, we acknowledge that the proportion of more severely affected individuals was comparatively low. We also did not include individuals with low IQ (< 50), which is a common limitation in imaging studies where experimental demands are too burdening for low-functioning individuals. This restriction compromises the heterogeneity of the LEAP cohort and its representativeness for the ASD population. Regarding our experimental protocol, this task uses graded levels of mental state attribution as a correlate for ToM, which might be less sensitive to capture specific effects of mentalizing on the neural and behavioral level. Small methodological differences to the original study by Castelli et al. [7] might have contributed to the lack of case-control differences on the behavioral level (e.g., use of 3-point instead of 6-point scale for obtaining narrative scores). Overall, the pattern of strong within-subject effects and small-to-absent between-subject effects might partly result from substantial between-subject heterogeneity, which may be exacerbated in multicenter designs, but is a feature of samples that strive for representativeness for the underlying population. While this is the case for the current study, we followed recommended procedures to ensure optimal alignment of data acquisition between centers [66] and aimed for a deep multimodal characterization of our study participants [24, 37]. Furthermore, our distributional observations do not support the interpretation of distinct subgroups of participants with ASD masking group mean effects.

## Conclusions

In the current report, we examined social brain correlates of mentalizing in ASD in a large and well-powered task-based fMRI dataset. Developmental effects were observed in younger individuals who showed a stronger modulation of attention-related brain areas by mentalizing demands. Against our expectation, we did not replicate previous observations of reduced activation in individuals with ASD. Instead, brain responses in autistic individuals were robustly typical in that they showed the expected effects of task in the social brain circuitry. We therefore conclude that time-locked functional activation in an animated shapes task does not inform the neurobiological basis of the mind-blindness account of ASD. This calls for the interrogation of different neural phenotypes, in particular connectivity and connectomic measures such as those derived from graph theory, which may access brain functional interaction not captured by the activation measures investigated here. Furthermore, our work clearly demonstrates the importance of novel research strategies that go beyond case-control comparisons but rather target the heterogeneity in ASD itself (e.g., [65]), an opportunity that is offered by large-scale data sets such as LEAP.

## Supplementary information

**Additional file 1.** Supplementary Material.

## Data Availability

The datasets generated and/or analyzed during the current study are not publicly available due to an embargo period but are available from the corresponding author on reasonable request.
