# Supplementary material for: Social brain activation during mentalizing in a large autism cohort: the Longitudinal European Autism Project
Source: Mol Autism. 2020 Feb 22;11:17. doi: 10.1186/s13229-020-0317-x (PMC7036196; doi:10.1186/s13229-020-0317-x)
Supplement: Supplementary file 1 — Additional file 1. Supplementary Material. [file 13229_2020_317_MOESM1_ESM.docx]

Supplemental Material

Contents

[1. Sample description by age group 2](#_Toc21971649)

[2. Standard operation procedures and quality control procedures 4](#_Toc21971650)

[2.1 fMRI 4](#_Toc21971651)

[2.2 Narratives 5](#_Toc21971652)

[3. Task description 6](#_Toc21971653)

[4. Behavioral task performance 7](#_Toc21971654)

[5. Activation analysis: results table, illustration of parametric effects and unthresholded T-map 8](#_Toc21971655)

[6. Control analyses 10](#_Toc21971656)

[6.1 Single contrasts 11](#_Toc21971657)

[6.2 IQ 12](#_Toc21971658)

[6.3 Site 13](#_Toc21971659)

[6.4 Behavioral performance 17](#_Toc21971660)

[6.5 Medication 18](#_Toc21971661)

[6.6 Motion and SNR 21](#_Toc21971662)

[6.7 Comorbidity (depression, anxiety, ADHD) 22](#_Toc21971663)

[6.8 Narrow ASD definition 26](#_Toc21971664)

[6.9 SRS self-report 28](#_Toc21971665)

[7. Effect of sex 29](#_Toc21971666)

[8. Association between video categorization and dmPFC activation 30](#_Toc21971667)

[9. References 31](#_Toc21971668)

1. Sample description by age group

Table S1: Participant characteristics, split by sample and age group.

|  | **youth sample** | | | |  | **adult sample** | |  |
| --- | --- | --- | --- | --- | --- | --- | --- | --- |
|  | ASD | | TD | | statistics | ASD | TD | statistics |
|  | children | adolescents | children | adolescents | TD vs. ASD | adults | adults | TD vs. ASD |
| total n | 30 | 81 | 32 | 73 |  | 94 | 84 |  |
| **Demographics** |  |  |  |  |  |  |  |  |
| sex (male/female) | 24/6 | 61/20 | 19/13 | 48/25 | χ^2^(1)=4.22, p=.040 | 66/28 | 56/28 | χ^2^(1)=.26, p=.611 |
| age (years) | 10.0 $\pm$ 1.5  (7.1 - 12.0) | 15.2 $\pm$ 1.7  (12.4 – 17.9) | 10.3 $\pm$ 1.2  (7.6 – 11.7) | 15.5 $\pm$ 1.7  (12.4 – 18.0) | t(214)=.46, p=.644 | 22.7 $\pm$ 3.5  (18.0 – 30.6) | 23.2 $\pm$ 3.2  (18.1 – 31.0) | t(176)=.91, p=.366 |
| IQ (full IQ) | 110.2 $\pm$ 13.8 (86 – 139) | 105.9 $\pm$ 14.6  (78.2 – 143.0) | 111.3 $\pm$ 11.0  (85 – 133) | 105.7 $\pm$ 12.2  (77 – 134) | t(212)=.20, p=.841 | 107.2 $\pm$ 14.1  (76 – 148) | 109.9 $\pm$12.0  (85 – 142) | t(176)=1.37, p=.173 |
| medication^1^  (% subjects) | 53.3 | 40.7 | 6.3 | 9.6 | χ^2^(1)=34.76, p<.001 | 34.0 | 2.4 | χ^2^(1)=28.78, p<.001 |
| medication^2^  antidepressants  antiepileptics  antimigraine  antipsychotics  anxiolytics  hypnotics/sedatives  opioids  other analgesics  psychostimulants | 0  1  0  2  2  10  0  0  6 | 2  0  0  8  0  13  0  1  20 | 0  0  0  0  0  1  1  0  0 | 1  0  0  0  0  1  0  2  4 |  | 20  3  0  3  0  6  1  2  6 | 0  0  1  0  0  0  0  2  0 |  |
| **In-scanner performance** |  |  |  |  |  |  |  |  |
| mean framewise displacement (FD; in mm)^3^ | .17 $\pm$ .08  (.06 - .34) | .15 $\pm$ .09  (.03 - .47) | .17 $\pm$ .11  (.06 -.42) | .13 $\pm$ .07  (.05 - .38) | t(214)=1.16, p=.249 | .12 $\pm$ .07  (.04 - .35) | .10 $\pm$ .06  (.03 - .30) | t(176)=1.87, p=.063 |
| volumes with FD > 0.5 mm (%) | 4.85 $\pm$ 5.09  (0 - 19.40) | 4.28 $\pm$ 5.10  (0 – 19.27) | 5.39 $\pm$ 5.81  (0 – 19.67) | 3.55 $\pm$ 4.41  (0 – 19.73) | t(214)=.48, p=.631 | 2.49 $\pm$ 4.08  (0 – 19.33) | 1.99 $\pm$ 3.10  (0 – 12.29) | t(176)=.91, p=.363 |
| signal-to-noise ratio | 11.23 $\pm$ .72  (10.22 - 13.60) | 9.64 $\pm$ 1.22  (6.65 – 12.93) | 11.63 $\pm$ 1.15  (8.28 – 13.77) | 9.73 $\pm$ 1.22  (6.50 – 12.18) | t(214)=1.27, p=.204 | 9.36 $\pm$ 1.18  (6.46 – 13.84) | 9.46 $\pm$ 1.12  (7.04 – 11.80) | t(176)=.63, p=.528 |
| task accuracy | .72 $\pm$ .17  (.33 - .92) | .82 $\pm$ .13  (.42 – 1.0) | .75 $\pm$ .18  (.33 – 1.0) | .85 $\pm$ .10  (.42 – 1.0) | t(214)=1.30, p=.194 | .84 $\pm$ .11  (.42 – 1.0) | .85 $\pm$ .13  (0 – 1) | t(176)=.52, p=.605 |
| **Clinical characteristics** |  |  |  |  |  |  |  |  |
| ADI-R^4^ |  |  |  |  |  |  |  |  |
| social interaction | 13.6 $\pm$ 6.7  (1 – 25) | 16.9 $\pm$ 6.6  (2 – 29) |  |  |  | 14.9 $\pm$ 6.7  (0 – 28) |  |  |
| communication | 12.2 $\pm$ 5.2  (3 – 22) | 13.2 $\pm$ 5.7  (1 - 26) |  |  |  | 12.1 $\pm$ 5.7  (0 - 24) |  |  |
| RRB | 4.5 $\pm$ 3.1  (0 – 12) | 4.1 $\pm$ 2.7  (0 - 12) |  |  |  | 3.7 $\pm$ 2.4  (0 – 12) |  |  |
| ADOS-2^5^ |  |  |  |  |  |  |  |  |
| social affect | 5.5 $\pm$ 2.3  (1 – 9) | 6.1 $\pm$ 2.6  (1 – 10) |  |  |  | 5.5 $\pm$ 2.4  (1 – 10) |  |  |
| RRB | 4.6 $\pm$ 3.0  (1 – 9) | 4.7 $\pm$ 2.3  (1 – 10) |  |  |  | 4.8 $\pm$ 2.7  (1 – 10) |  |  |
| total | 4.6 $\pm$ 2.3  (1 – 9) | 5.3 $\pm$ 2.7  (1 – 10) |  |  |  | 4.8 $\pm$ 2.5  (1 – 10) |  |  |
| SRS (parent report)^6^ |  |  |  |  |  |  |  |  |
| raw score | 93.4 $\pm$ 32.6  (32 – 163) | 92.6 $\pm$ 28.0  (33 – 151) | 19.5 $\pm$ 14.7  (2 – 74) | 20.1 $\pm$ 14.7  (1 – 74) | t(174)=20.65 p<.001 | 77.0 $\pm$ 30.2  (21 – 143) | *n.a.* |  |
| T score | 73.8 $\pm$ 12.0  (49 – 90) | 73.9 $\pm$ 10.6  (50 – 90) | 45.0 $\pm$ 5.7  (37 – 66) | 45.2 $\pm$ 6.0  (37 – 66) | t(174)=21.29p<.001 | 62.9 $\pm$ 10.6  (43 – 86) | *n.a.* |  |
| DAWBA comorbidities^7^ |  |  |  |  |  |  |  |  |
| ADHD symptoms | 2.2 $\pm$ 1.5  (0 – 5) | 2.1 $\pm$ 1.6  (0 – 5) | 0.4 $\pm$ 0.9  (0 – 3) | 0.1 $\pm$ 0.5  (0 – 3) | t(155)=9.47,  p<.001 | 0.9 $\pm$ 1.3  (0 – 4) | *n.a.* |  |
| Depression symptoms | 1.1 $\pm$ 1.4  (0 – 5) | 1.0 $\pm$ 1.2  (0 – 4) | 0 $\pm$ 0.2  (0 – 1) | 0.5 $\pm$ 0.8  (0 – 4) | t(175)=4.29,  p<.001 | 1.1 $\pm$ 1.4  (0 – 5) | 0.4 $\pm$ 0.7  (0 – 3) | t(175)=3.68,  p<.001 |
| Anxiety symptoms | 2.4 $\pm$ 1.4  (1 – 5) | 2.5 $\pm$ 1.4  (0 – 5) | 1.0 $\pm$ 0.7  (0 – 3) | 1.0 $\pm$ 0.9  (0 – 4) | t(186)=8.55,  p<.001 | 2.6 $\pm$ 1.3  (0 – 4) | 2.4 $\pm$ 0.8  (0 – 4) | t(160)=7.00,  p<.001 |

^1^Percentage of participants taking medication prescribed for behavioral or neurological problems. Medication data was available for 74 ASD and 50 TD participants in the youth sample, and 58 ASD and 28 TD participants in the adult sample. ^2^Number of participants taking specific classes of medication (antidepressants: selective serotonin reuptake inhibitor, tricyclic antidepressant, tetracyclic antidepressant; antipsychotics: aripiprazole, clozapine, pipamperon, quetiapine, risperidone; hypnotics and sedatives: hyoscine butylbromide, melatonin, niaprazine, temazepam; psychostimulants and other drugs used to treat ADHD: atomoxentine, dexamfetamine, methylphenidate hydrochloride). ^3^Motion was assessed as mean framewise displacement according to Jenksinson et al. (2002). ^4^Autism Diagnostic Interview-Revised (ADI-R; (1)). Scores were computed for reciprocal interaction (social interaction), communication, and restrictive, repetitive stereotyped behaviors and interests (RRB). ADI-R scores were available for 108 ASD participants in the youth sample, and 89 ASD participants in the adult sample. ^5^Autism Diagnostic Observation Schedule 2 (ADOS-2;(2)). Calibrated severity scores were computed for social affect, restricted and repetitive behaviors (RRB) and the overall total score. ADOS scores were available for 107 ASD participants in the youth sample, and 91 ASD participants in the adult sample. ^6^Total raw and total T score (sex and age normalized) on the Social Responsiveness Scale-2 (SRS-2; (3)). SRS-2 scores were available for 176 participants in the youth sample, and 71 participants in the adult sample. The raw SRS-2 scores were used in our analyses. ^7^Comorbid symptoms of ADHD, depression and anxiety were assessed with the Development and Well Being Assessment (DAWBA; (4)), generating six levels (ordinal scores 0 to 5) of prediction of the probability of a disorder (~0.1%, ~0.5%, ~3%, ~15%, ~50%, >70%). DAWBA scores for depression were available for 93 ASD and 84 TD participants in the youth sample, and 74 ASD and 62 TD participants in the adult sample. DAWBA scores for ADHD were available for 84 ASD and 73 TD participants in the youth sample. DAWBA scores for anxiety were available for 96 ASD and 92 TD participants in the youth sample, and 87 ASD and 75 TD participants in the adult sample.

1. Standard operation procedures and quality control procedures
   1. fMRI

Standard operation procedures were implemented to mitigate differences in data acquisition between sites and across time (5). This included hands-on training according to detailed protocols before study rollout and regular exchange between sites. Hard- and software for data acquisition was aligned as closely as possible between sites. Test-retest reliability of the fMRI task battery was ensured (6-8).

After excluding datasets due to brain abnormality (N=8), incomplete task coverage (N=5), or technical reasons (N=27), a total of 467 datasets were subjected to fMRI data analysis and to an extensive fMRI data quality assessment (QA) pipeline. Several QA metrics were calculated (<http://preprocessed-connectomes-project.org/quality-assessment-protocol/>). Head motion was quantified as frame-wise displacement (FD, in mm; 9). Two aggregate scores were derived for each dataset: 1) mean FD and 2) percent of volumes exceeding 0.5 mm FD (percFD_05; lower values are better). Additional temporal QA metrics (i.e. QA metrics pertaining to the temporal evolution of the 3D volume) included the 3) temporal signal-to-noise ratio (tSNR; higher values are better) and the 4) average change of a volume’s mean intensity across time points (DVARS (10); lower values are better). We additionally calculated the 5) signal-to-noise ratio (SNR, higher values are better) as a spatial QA metric based on the mean volume of the realigned time-series. Here, the mean signal intensity in the foreground region is divided by the standard deviation of the signal intensity in the background region.

Since motion is the primary source of bad data quality, subjects with more than 20 % of frames exceeding 0.5 mm FD were excluded (ASD: n = 47, 19 %; TD: n = 26, 12 %). This quality control step effectively addressed other effects of low data quality, such as excessive signal loss due to bad brain coverage (as assessed as overlap between individual brain mask and MNI template brain, in percent, before QC: [73.3 94.9], after QC: [83.0 94.9]). A comparison of main QA parameters between TD and ASD subjects before and after QC is depicted in table S2.

**Table S2**: Group differences in quality parameters before and after adjusting the sample for motion outliers

|  | **initial sample** | |  | **final sample** | |  |
| --- | --- | --- | --- | --- | --- | --- |
|  | **ASD** | **TD** | **statistics** | **ASD** | **TD** | **statistics** |
| N | 252 | 215 |  | 205 | 189 |  |
| mean FD | .28  (.40) | .22  (.30) | t_(459)_* = 2.039  *p* = .042 | 0.14 (0.08) | .12  (.08) | t_(392)_ = 1.877,  *p* = .061 |
| percFD_05 | 10.80 (17.32) | 7.66  (13.90) | t_(463)_* = 2.175  *p* = .030 | 3.54 (4.73) | 3.16  (4.33) | t_(392)_ = .822,  *p* = .412 |
| tSNR | 55.96 (20.16) | 60.03 (20.33) | t_(465)_ = 2.168  *p* =.031 | 61.79 (17.52) | 64.21 (17.85) | t_(392)_ = 1.359,  *p* = .175 |
| DVARS | 1.18  (.14) | 1.17  (.15) | t_(465)_ = .219  *p* = .827 | 1.19 (0.14) | 1.18  (0.15) | t_(392)_ = .184,  *p* = .854 |
| SNR | 10.07 (1.51) | 10.19 (1.69) | t_(465)_ = .813  *p* = .417 | 9.74 (1.30) | 9.93  (1.39) | t_(392)_ = 1.424,  *p* = .155 |

* statistic corrected for unequal variance

- 1. Narratives

The acquisition and analysis of narratives was performed by trained personnel and followed standard operation procedures. The recording of the narratives required minimal intervention, with one standardized instruction and minimal interaction during recording. The analysis of the narratives was performed by two trained raters at each site and followed a strict coding scheme. In contrast to the original 5-point scale in Castelli and colleagues (11), we developed a 3-point scale in order to a) improve standardization and reliability, and to b) create a more robust and stringent coding process with improved discrimination between high and low performers. An inter-rater reliability analysis using Kappa statistic was performed to determine consistency among raters, which was found to be excellent (κ=0.907).

1. Task description

The animations were identical to those used in the study of Castelli and colleagues (11)(see <https://sites.google.com/site/utafrith/research> for exemplary video clips). When originally designed, each clip was assigned to one of the three conditions (Theory of Mind [ToM], Goal-Directed [GD], Random) based on ratings of an adult TD sample (12). ToM animations follow a ‘script’ where one triangle aims at manipulating the mental state of the other triangle (coaxing, surprising, mocking, seducing). GD animations depict purposeful physical interactions between both triangles, which conveys the perception of agency (leading, dancing, fighting, chasing). In Random animations, triangles show self-propelled motion without any interaction (bouncing or floating about the screen).

fMRI: Contrary to Castelli et al. (11), the fMRI task followed an updated method where participants are not instructed to give verbal descriptions after each video clip (prompted by the question: “What was happening in this animation?”) but instead are asked to categorize each animation in a multiple choice manner. This method has been validated as an objective test to assess on-line mentalizing in a sample of adult participants with ASD and TD controls, and comes with the advantage of a faster assessment and more objective analysis of the participant’s response (13).

Participants were introduced to the three conditions in a practice session outside the scanner using one exemplary animation per condition (not shown during fMRI scanning). Random animations were described as video clips where “both triangles move across the screen on their own, independently from each other”. GD animations were described as video clips where “the movement of one triangle is somehow related to the movement of the other triangle, such as playing ball or racing against each other”. ToM animations were described as video clips where “both triangles appear to think about the feelings and thoughts of the other triangle, or where one triangle is trying to convince the other triangle to do something”. Each condition was assigned to a representative icon (see Figure S1), and participants were trained to press the corresponding button on a response device after each practice trial.

The trial structure of the fMRI task is depicted in Figure S1.


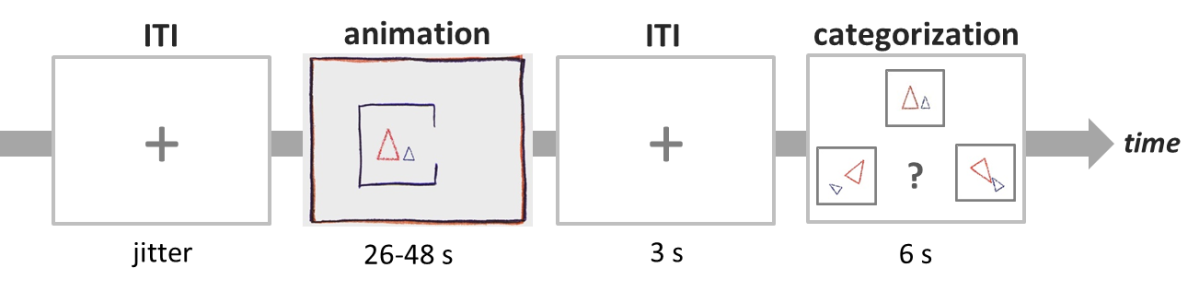


**Figure S1**: Trial structure of the fMRI animated shapes task. The jitter preceded each animation with a variable duration (M = 996 ms, SD = 418). The overall duration of the fMRI task was 10.3 minutes. ITI intertrial interval.

**Narratives:** Participants watched the same animations (except for two random animations) in a separate test session. Animations were presented in a pseudorandomized order. After a standardized instruction, participants verbalized continuously what was going on in the animation and responses were audio-typed. Interaction with the experimenter was kept on a minimal level and was limited to simple instructions (e.g. “ok, and what happens next?” when the subject stops talking or asks for feedback).

1. Behavioral task performance

In line with the fMRI data analyses, task performance during the fMRI scan was additionally evaluated in both age-specific subsamples (youth sample, adult sample). Analyses were performed in SPSS (IBM, version 22) using univariate Analyses of Covariance to assess the effects of diagnosis and age while controlling for sex, site and IQ.

We did not observe any main effect of diagnosis (all *F* ≤ 2.586, *p* ≥ .109). A significant diagnosis x age interaction emerged in the adult sample (*F*=5.236, *p*=.023), which needs to be interpreted with caution due to the skewed distribution of the data (Saphiro-Wilk test, *W* = .856, *p* < .001; Figure S2). In contrast, an effect of age was apparent in the full and youth samples, with better performance in older subjects (*F* = 20.220, p<.001; Figure S2). For details, see Table S3.

**Table S3**: Video categorization accuracy for the full sample, youth sample (< 18 years of age) and adult sample ($\geq$18 years of age)

|  | **ASD** | **TD** | **ME diagnosis** | **ME age** | **IA age x diagnosis** |
| --- | --- | --- | --- | --- | --- |
| overall categorization accuracy (in %) | | |  |  |  |
| full sample | 81.4 $\pm$ 13.4 | 83.2 $\pm$ 13.4 | *F*_(1,381)_=.383  *p*=.536 | ***F*_(1,381)_=20.220**  ***p*<.001** | *F*_(1,381)_=.727  p=.394 |
| youth sample | 79.4 $\pm$14.6 | 81.9 $\pm$ 13.7 | *F*_(1,204)_=.265  *p*=.608 | ***F*_(1,204)_=36.133**  ***p*<.001** | *F_(_*_1,203)_=.112  *p*=.739 |
| adult sample | 83.8 $\pm$ 11.4 | 84.7 $\pm$ 13.0 | *F*_(1,167)_=.338  *p*=.562 | *F*_(1,167)_=3.355  *p*=.069 | ***F*_(1,166)_=5.236**  ***p*=.023** |
| ToM video categorization accuracy (in %) | | |  |  |  |
| full sample | 82.7 $\pm$ 20.5 | 86.2 $\pm$ 19.0 | *F*_(1,381)_=2.181  *p*=.141 | ***F*_(1,381)_=28.377**  ***p*<.001** | *F*_(1,381)_=.097  *p*=.755 |
| youth sample | 78.8 $\pm$ 21.4 | 83.8 $\pm$ 19.6 | *F*_(1,204)_=2.586  *p*=.109 | ***F*_(1,204)_=39.411**  ***p*<.001** | ***F***_(1,203)_=.732  *p*=.393 |
| adult sample | 87.2 $\pm$ 18.6 | 89.3 $\pm$ 17.9 | *F*_(1,167)_=.441  *p*=.508 | *F*_(1,167)_=.696  *p*=.405 | *F*_(1,166)_=1.990  *p*=.160 |

Significant effects printed in bold; ASD autism spectrum disorder, TD typical development, ME main effect, IA interaction effect

**
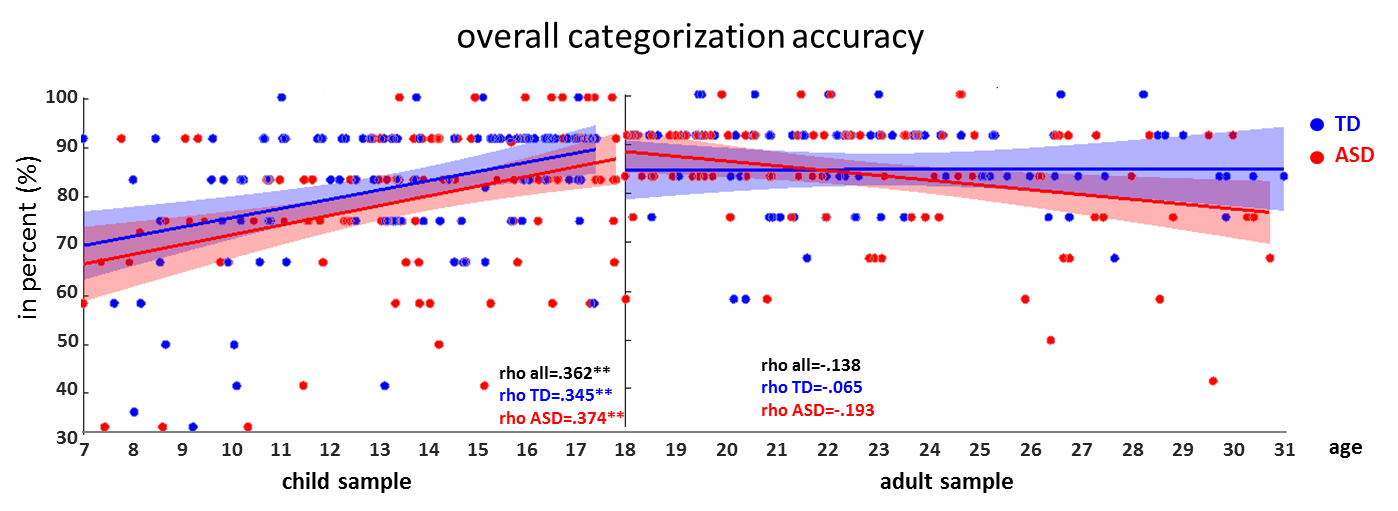
**

**Figure S2:** Overall categorization accuracy. Linear least square regression fits for TD (blue) and ASD (red) are shown for the youth sample (< 18 years of age) and adult sample (≥ 18 years of age). Shaded areas represent 95% confidence intervals. Non-parametric correlation coefficients (Spearman’s rho) are reported for all subjects (black) and separately for TD subjects (blue) and ASD subjects (red).* *p*<.05, ** *p*<.001.

1. Activation analysis: illustration of parametric effects and unthresholded T-map


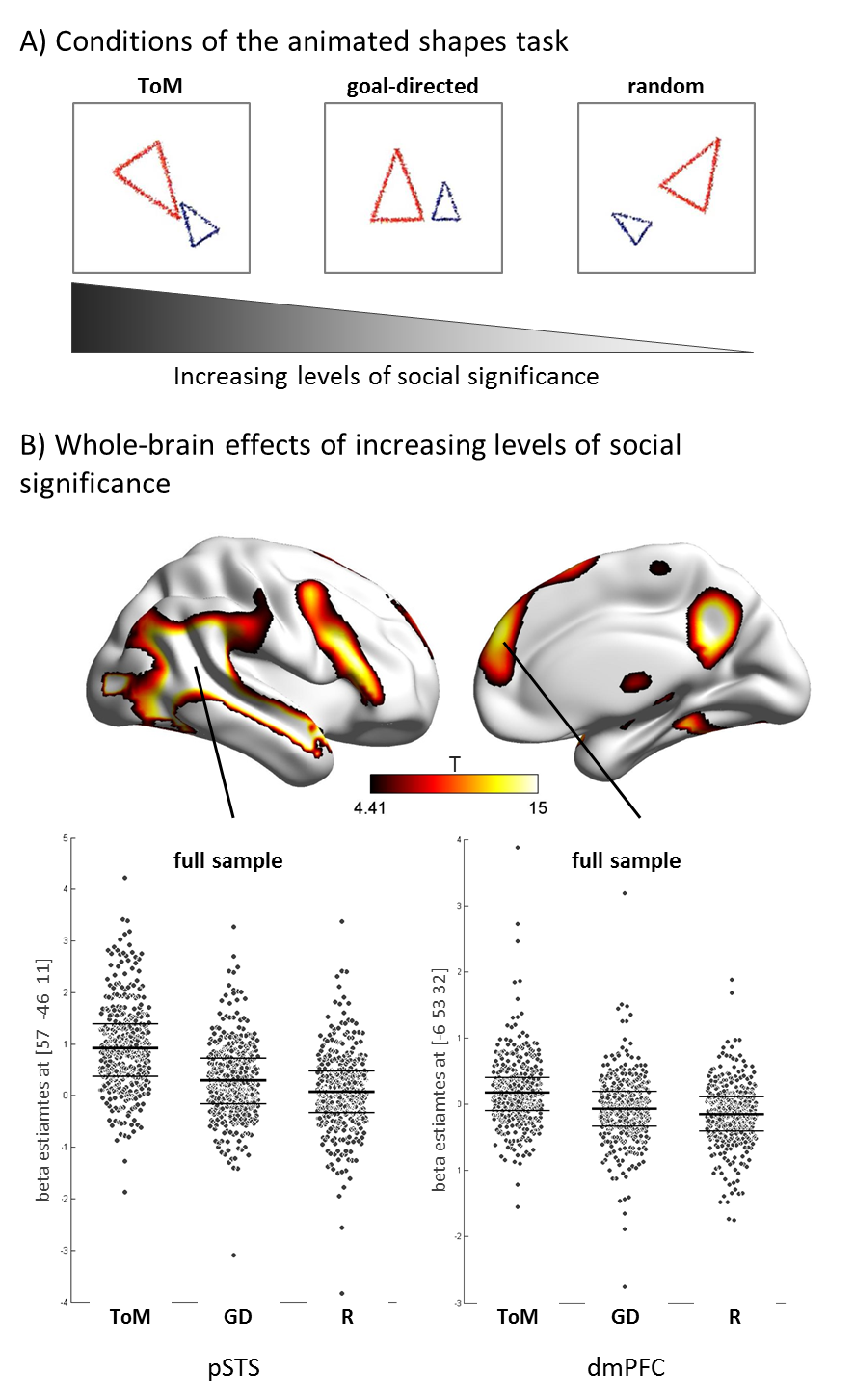


**Figure S3:** A) Brain activation was elicited by animated video clips featuring two triangles whose movement patterns reflected increasing levels of social significance according to three conditions: 1) Random movement (R; no interaction,) 2) goal-directed movement (GD, simple interactions, e.g. chasing), and 3) Theory of Mind (ToM; complex interactions,e.g. cheating). B) Whole-brain effects were calculated using a parametric modulation of increasing levels levels of mental state attribution (ToM > GD > R), as decribed in the main text. This parametric effect is reflected by the distribution of beta weights pertaining to each of the three conditions for peak voxels within the two regions of interest (posterior superior temporal sulcus, pSTS; dorsomedial prefrontal cortex, dmPFC). Note that these beta estimates were generated in a separate model where each condition was modeled separately, i.e. without parametric modulation.


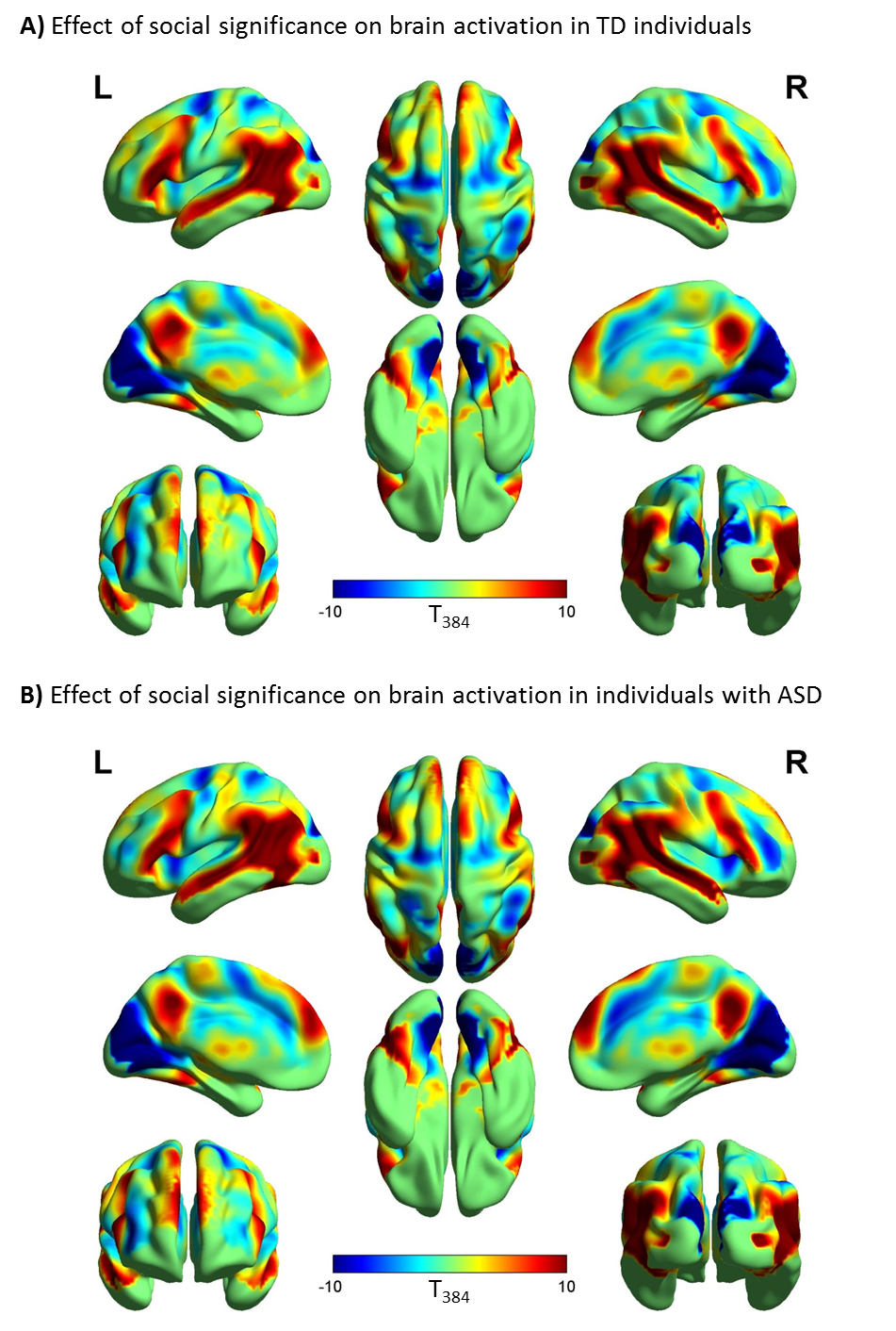


**Figure S4**: Effect of parametric modulation of social significance on brain activation in (A) TD individuals (n=189) and (B) individuals with ASD (n=205), illustrated as unthresholded SPM-T maps rendered on a standard brain (ICBM152). Effects are visualized with the BrainNet Viewer (http://www.nitrc.org/projects/bnv/).

1. Control analyses

Several control analyses were conducted to 1) ensure that the overall lack of case-control effects on brain activation is not a consequence of unaddressed sources of variance, and to 2) test the robustness of the observed positive association between autism traits and dmPFC activation. In these analyses, effects were corrected for demographic variables (IQ, site), task performance (overall categorization accuracy), clinical characteristics (medication status, presence of comorbidities), or functional image quality (motion, signal-to-noise ratio). In addition, we repeated the analyses in a restricted sample with individuals that meet more conservative criteria of ASD diagnosis (narrow ASD definition). We also tested whether dimensional effects of autism traits persisted when using scores derived from the SRS self-report, which was available for adult individuals (TD and ASD) as well as for adolescent TD individuals. Finally, in order to align more closely with the analyses performed in previous work (e.g., 6, 11, 14), we repeated the analyses where the task condition of interest was contrasted to the high- and low-level baseline conditions, respectively (i.e. ToM > GD, ToM > R). All analyses were performed for the full sample, youth sample and adult sample, if applicable.

Given the high number of tests, we restrict our report to the test statistic of significant peak voxels and – in case of no significance – of the overall peak voxel of the respective test. Voxel-level statistics were family-wise error corrected for the number of voxels within the search masks (whole brain or small-volume correction within the combined mask of dmPFC and right pSTS) for each test. Given our aim to maximize sensitivity, no correction for multiple comparisons was applied for the total number of control analyses. For easier assessment of the results tables below, deviations from the outcome reported in the main text are highlighted in bold.

- 1. Single contrasts

In previous studies, including Castelli et al. (11), ToM-specific brain responses were defined by contrasting the experimental condition to the low- or high-level baseline condition. Our main analyses, in contrast, were based on a parametric modeling of mentalizing demands across all three experimental conditions. For replication purposes and to rule out that the lack of case-control differences was due to a different modeling of mentalizing-related brain responses, we here report supplementary statistical information on the single-contrast results (ToM > Random, ToM > Goal-directed) presented in the main text.

**Table S4:** Overview over effects of diagnosis (categorical) and autism traits (dimensional) on functional brain activation as defined by the contrasts ToM > random and ToM > goal-directed.

|  | **TD vs. ASD (categorical)** | | | **positive association with autism traits (dimensional)** | | |
| --- | --- | --- | --- | --- | --- | --- |
|  | full sample | youth sample | adult sample | full sample (no TD adult^1^) | youth sample | adult sample (ASD only^1^) |
| original results (reported in main text) | no effect | no effect | no effect | positive association with inferior parietal cortex (whole brain) and dmPFC (ROI) | positive association with dmPFC (ROI) | no effect |
| **ToM > random** | |  |  |  |  |  |
| whole-brain | no effect (all F_(1,384)_≤12.40, p_FWE_≥.860) | no effect (all F_(1,207)_≤12.47, p_FWE_≥.887) | no effect (all F_(1,168)_≤18.13, p_FWE_≥.277) | no effect (all t_(236)_≤4.50, p_FWE_≥.069) | **t_(166)=_4.92, p_FWE_=.017 at [-54 26 11] in inferior frontal gyrus (pars triangularis)** | no effect (all t_(57)_≤3.74, p_FWE_≥.789) |
| right pSTS | no effect (all F_(1,384)_≤3.58, p_FWE_≥.911) | no effect (all F_(1,207)_≤8.08 p_FWE_≥.294) | no effect (all F_(1,168)_≤7.50, p_FWE_≥.420) | no effect (all t_(236)_≤2.67, p_FWE_≥.292) | no effect (all t_(166)_≤2.90, p_FWE_≥.179) | no effect (all t_(57)_≤1.82, p_FWE_≥.809) |
| dmPFC | no effect (all F_(1,384)_≤3.43, p_FWE_≥.924) | no effect (all F_(1,207)_≤4.82, p_FWE_≥.831) | no effect (all F_(1,168)_≤4.72, p_FWE_≥.887) | t_(236)=_3.35, p_FWE_=.057 at [3 62 23] | t_(166)=_4.20, p_FWE_=.004 at [-15 50 35] | no effect (all t_(57)_≤2.07, p_FWE_≥.678) |
| **ToM > goal-directed** | |  |  |  |  |  |
| whole-brain | no effect (all F_(1,384)_≤13.92, p_FWE_≥.690) | no effect (all F_(1,207)_≤17.35, p_FWE_≥.298) | no effect (all F_(1,168)_≤14.13, p_FWE_≥.747) | **no effect (all t_(236)_≤3.76, p_FWE_≥.540)** | no effect (all t_(166)_≤3.67, p_FWE_≥.658) | **t_(57)_=5.32, p_FWE_=.016 at [-36 14 50] in left middle frontal gyrus** |
| right pSTS | no effect (all F_(1,384)_≤3.12, p_FWE_≥.956) | no effect (all F_(1,207)_≤4.19, p_FWE_≥.867) | no effect (all F_(1,168)_≤5.52, p_FWE_≥.690) | no effect (all t_(236)_≤2.66, p_FWE_≥.282) | no effect (all t_(166)_≤2.10, p_FWE_≥.641) | no effect (all t_(57)_≤2.40, p_FWE_≥.451) |
| dmPFC | no effect (all F_(1,384)_≤8.57, p_FWE_≥.251) | **F_(1,207)_≤13.56, p_FWE_≥.034 at [9 50 26]** | no effect (all F_(1,168)_≤2.84, p_FWE_≥.973) | **no effect (all t_(236)_≤2.65, p_FWE_≥.291)** | **no effect (all t_(166)_≤1.88, p_FWE_≥.772)** | **t_(57)=_3.51, p_FWE_=.048 at [-9 47 41]** |

^1^ no parent-reported SRS-scores available for TD adults;

Table gives test statistic of significant peak voxel(s) or, in case of no significance, of the overall peak voxel of the respective test. Voxel-level statistics were family-wise error corrected for the number of voxels within the search masks (whole brain or small-volume correction within the combined mask of dmPFC and right pSTS) for each test. Deviations from results reported in the main text are highlighted in bold. Significant whole-brain results are localized in MNI space and labeled according to the automated anatomical labeling atlas (aal). Abbreviations: TD typically developing, ASD autism spectrum disorder, pSTS posterior superior temporal sulcus, dmPFC dorsomedial prefrontal cortex, ROI region of interest.

- 1. IQ

Level of intellectual abilities was assessed using the Wechsler Abbreviated Scales of Intelligence – Second Edition, WASI-II (15) or – in countries where the WASI is not translated (i.e. The Netherlands, Germany and Italy) – the four-subtest short-forms of the German, Dutch or Italian WISC-III/IV ((16, 17) for children) or WAIS-III/IV ((18, 19) for adults). The shortened versions were used for feasibility reasons to not further prolong the testing sessions for participants. All versions included two verbal subscales (Vocabulary, Similarities) and two non-verbal subscales (Block Design, Matrix Reasoning). Full-scale IQ was pro-rated from subscales using an algorithm developed by Sattler (1992) that produces an estimated IQ score that is highly correlated (*r* = .93) with a Full-Scale IQ obtained by administering the complete test. Age-appropriate national population norms were available for each participating site and these were used to derive standardized estimates of an individual’s intellectual functioning. Where recent IQ scores from previous assessments were available (less than 12 months in children; less than 18 months in adolescents and adults) IQ tests were not repeated.

IQ scores were added as additional covariate of no interest in the sample-specific second-level GLMs on brain activation. Results do not suggest any influence of IQ on the autism-related effects across samples and search regions (whole brain, combined dmPFC and right pSTS ROI; table S8).

**Table S5**: Overview over effects of diagnosis (categorical) and autism traits (dimensional) on functional brain activation after controlling for intellectual ability.

|  | **TD vs. ASD (categorical)** | | | **positive association with autism traits (dimensional)** | | |
| --- | --- | --- | --- | --- | --- | --- |
|  | full sample | youth sample | adult sample | full sample (no TD adult^1^) | youth sample | adult sample (ASD only^1^) |
| original results (reported in main text) | no effect | no effect | no effect | positive association with inferior parietal cortex (whole brain) and dmPFC (ROI) | positive association with dmPFC (ROI) | no effect |
| whole-brain | no effect (all F_(1,381)_≤12.60, p_FWE_≥.909) | no effect (all F_(1,204)_≤12.58, p_FWE_≥.901) | no effect (all F_(1,167)_≤18.36, p_FWE_≥.260) | no effect (all t_(234)_≤4.56, p_FWE_≥.058) | no effect (all t_(164)_≤4.54, p_FWE_≥.073) | no effect (all t_(56)_≤4.31, p_FWE_≥.290) |
| right pSTS | no effect (all F_(1,381)_≤3.37, p_FWE_≥.954) | no effect (all F_(1,204)_≤8.03, p_FWE_≥.314) | no effect (all F_(1,167)_≤8.94, p_FWE_≥.257) | no effect (all t_(234)_≤2.92, p_FWE_≥.181) | no effect (all t_(164)_≤2.98, p_FWE_≥.155) | no effect (all t_(56)_≤2.04, p_FWE_≥.685 |
| dmPFC | no effect (all F_(1,381)_≤4.71, p_FWE_≥.825) | no effect (all F_(1,204)_≤7.93, p_FWE_≥.326) | no effect (all F_(1,267)_≤3.48, p_FWE_≥.950) | t_(234)_=3.73, p_FWE_=.019 at [3 62 23] | t_(164)=_3.72, p_FWE_=.020 at [3 56 26] and t_(164)_=3.44, p_FWE_=.047 at [-15 50 35] | no effect (all t_(56)_≤2.97, p_FWE_≥.173) |

^1^ no parent-reported SRS-scores available for TD adults;

Table gives test statistic of significant peak voxel(s) or, in case of no significance, of the overall peak voxel of the respective test. Voxel-level statistics were family-wise error corrected for the number of voxels within the search masks (whole brain or small-volume correction within the combined mask of dmPFC and right pSTS) for each test. Deviations from results reported in the main text are highlighted in bold. Significant whole-brain results are localized in MNI space and labeled according to the automated anatomical labeling atlas (aal). Abbreviations: TD typically developing, ASD autism spectrum disorder, pSTS posterior superior temporal sulcus, dmPFC dorsomedial prefrontal cortex, ROI region of interest.

- 1. Site

The influence of site was investigated by recalculating the statistical maps while holding out one site at a time (leave-one-out), thereby preserving statistical power when examining systematic effects of site.

Results do not suggest a systematic effect of site on case-control differences (table S9). However, the association between dmPFC activation and dimensional autism traits turned insignificant when dropping one site (KCL) in the full and youth samples ((1) full sample: KCL subjects n=113, association at MNI coordinate [3 62 23] with KCL subjects: Beta=.338, p=.001 (multiple regression, F(9,237)=3.206, p<.001, R2=.11); without KCL subjects: Beta=.090, p=.467 (multiple regression, F(8,168)=2.441, p=.016, R2=.10); (2) youth sample: KCL subjects n=48; association at MNI coordinate [3 56 26] with KCL subjects: Beta=.476, p=.001 (multiple regression, F(8,167)=3.246, p=.002, R2=.14); without KCL subjects: Beta=.180, p=.256 (multiple regression, F(7,125)=2.532, p=.018, R2=.12)). In addition, the whole-brain effect of autism traits in the inferior parietal cortex similarly turned insignificant when excluding subjects from the KCL site, as well as when excluding subjects from the Cambridge site.

We performed follow-up analyses to better understand the KCL site effect on the association between dmPFC activation and autism traits. We considered three potential reasons for this effect: statistical reasons, hardware related reasons, and sample-related reasons.

Regarding potential statistical reasons, post-hoc power analyses show that the effect cannot be attributed to a loss of statistical power. With KCL subjects (full sample), the power is 0.9999997 (parameters: α=.05, n=394, effect size ƒ^2^=.122, number of predictors=9), without KCL (full sample), the power is .9999046 (parameters: α=.05, n=281, effect size ƒ^2^=.116, number of predictors=8). Likewise, the standard error of the estimate barely changed as a result of a change in N (with KCL: .44, without KCL: .43). The increase in sample size is therefore not a plausible explanation of the KCL site effect.

Potential hardware related reasons cannot be completely ruled out. However, the range of contrast estimates extracted from the dmPFC is comparable to that of other sites (see below). In addition, contrast estimates are a relative measures of brain activation (i.e. condition A vs. condition B), which largely controls for site-specific differences in absolute signal strength. We therefore assume that the KCL site effect is not a result of differences in imaging equipment.

We next tested for differences in sample characteristics. These analyses were restricted to subjects for whom a parent-reported SRS score was available. Results are as follows:

1. KCL subjects had no elevated levels of motion (mean frame-wise displacement as key parameter for data quality; KCL: M=.14, SD=.08; all other sites combined: M=.14, SD=.08; F(1,243)=.241, p=.624 [controlled for the effect of diagnosis and age]);
2. the KCL sample did not differ in dmPFC activation (i.e. contrast estimate), although values were descriptively lower (M=.20, SD=.48; all other sites combined: M=.31, SD=.45; F(1,342)=2.897, p=.090 [controlled for the effect of diagnosis, age and sex]);
3. the KCL sample had a higher proportion of adult subjects (42% youth, 58% adults; all other sites combined: 60% youth, 40% adults; Pearson Chi-Square=9.748, p=.002)
4. the KCL sample had a higher proportion of ASD subjects (19% TD, 81% ASD; all other sites combined: 42% TD, 58% ASD; Pearson Chi-Square=11.871, p=.001);
5. the KCL sample had higher SRS scores (M=81.17, SD=41.75; all other sites combined: M=55.51, SD=38.40; F(1,242)=9.799, p=.002 [controlled for the effect of diagnosis, age and sex]);
6. KCL individuals with ASD had more severe autism symptoms according to ADOS (RBB subscale: M=6.38, SD=2.16; all other sites combined: M=3.98, SD=2.47; F= 37.230, p<.001) and ADI-R (social domain (M=17.9, SD=6.6; all other sites combined: M=14.6, SD=6.4; F(1,153)=10.466, p=.001), communication domain (M=14.7, SD=5.9; all other sites combined: M=11.8, SD=5.2; F(1,153)=11.756, p=.001), and RBB domain (M=4.9, SD=2.3; all other sites combined: M=3.5, SD=2.7; F(1,153)=11.018, p=.001; all analyses were controlled for the effect of age and sex)

These results suggest that the KCL site effect is most plausibly explained by the fact that the KCL site contributed a (relatively) higher number of (relatively) more severely affected individuals with ASD, which boosted the association between dmPFC activation and autism trait scores (Figure S5).

**Table S6**: Overview over effects of diagnosis (categorical) and autism traits (dimensional) on functional brain activation after controlling for site using the leave-one-out approach.

|  | **TD vs. ASD (categorical)** | | | | | | **positive association with autism traits (dimensional)** | | | | | |  |
| --- | --- | --- | --- | --- | --- | --- | --- | --- | --- | --- | --- | --- | --- |
|  | full sample | | youth sample | | adult sample | | full sample (no TD adult^1^) | | | youth sample | | adult sample (ASD only^1^) |  |
| original results (reported in main text) | | no effect | | no effect | | no effect | | positive association with inferior parietal cortex (whole brain) and dmPFC (ROI) | positive association with dmPFC (ROI) | | no effect | | |
| whole-brain | no effect  (all F≤17.96, all p_FWE_≥.243) | | no effect  (all F≤19.02, all p_FWE_≥.173) | | no effect  (all F≤18.74, p_FWE_≥.244) | | drop site CIMH(1)^2^: t_(222)_=4.64, p_FWE_=.049 at [54 -55 38] in inferior parietal cortex  drop site CIMH(2)^2^: t_(218)_=5.21, p_FWE_=.003 at [54 -55 41] in inferior parietal cortex  drop site RUNMC: t_(150)_=4.76, p_FWE_=.033 at [57 -55 38] in inferior parietal cortex  **drop site UMCU**: t_(212)_=5.01, p_FWE_=.006 at [54 -55 38] in inferior parietal cortex and **t_(212)_=4.45, p=.048 at [42 -67 -19] in fusiform gyrus**  drop site UCBM: t_(232)_=4.75, p_FWE_=.017 at [57 -55 38] in inferior parietal cortex  **no effect for drop site UCAM: all t_(211)_≤3.83, p_FWE_≥.490**  **no effect for drop site KCL: all t_(167)_≤3.43, p_FWE_≥.828** | | | **drop site CIMH(1)** ^2^**: t_(153)_=4.66, p_FWE_=.030 at [-54 26 11] in inferior frontal gyrus (pars triangularis)** | | no effect  (t≤4.31, p_FWE_≥.320) |  |
| right pSTS | no effect  (all F≤4.96, p_FWE_≥.788) | | **drop site UMCU: F_(1,179)_=12.98, p_FWE_=.044 at [51 -43 23]** | | **drop site KCL: F_(1,104)_=14.76, p_FWE_=.031 at [48 -43 -4]** | | no effect  (all t_(212)_≤3.24, p_FWE_≥.055) | | | **drop site CIMH(1)** ^2^**: t_(153)_=3.33, p_FWE_=.047 at [60 -49 17]**  **drop site UCAM: t_(153)_=3.64, p_FWE_=.024 at [48 -31 5]** | | no effect  (all t≤2.44, p_FWE_≥.459) |  |
| dmPFC | no effect  (all F≤8.75, p_FWE_≥.258) | | no effect  (all F≤12.03, p_FWE_≥.070) | | no effect  (all F≤5.29, p_FWE_≥.765) | | drop site CIMH(1) ^2^: t_(222)_=3.59, p_FWE_=.032 at [3 62 23]  drop site CIMH(2) ^2^: t_(218)_=3.46, p_FWE_=.031 at [6 56 23]  drop site UCAM: t_(211)_=3.50, p_FWE_=.035 at [6 56 26]  drop site RUNMC: t_(150)_=3.78, p_FWE_=.016 at [3 62 23]  drop site UMCU: t_(212)_=3.68, p_FWE_=.014 at [3 62 23]  drop site UCBM: t_(232)_=3.48, p_FWE_=.029 at [6 56 26]  **no effect for drop site KCL: all t_(167)_≤2.35, p_FWE_≥.433** | | | drop site CIMH(1) ^2^: t_(153)_=3.90, p_FWE_=.008 at [3 56 26]  drop site CIMH(2) ^2^: t_(149)_=3.83, p_FWE_=.014 at [6 56 23]  drop site UCAM: t_(153)_=3.70, p_FWE_=.015 at [3 53 29]  drop site RUNMC: t_(98)_=3.84, p_FWE_=.013 at [3 62 23]  drop site UMCU: t_(149)_=4.01, p_FWE_=.006 at [3 62 23]  **no effect for drop site KCL: all t_(124)_≤3.22, p_FWE_≥.072** | | no effect  (all t≤2.82, p_FWE_≥.239) |  |

^1^ no parent-reported SRS-scores available for TD adults; ^2^ site CIMH was split into two sites (CIMH 1: n=23, CIMH 2: n=42) due to a difference in scanner settings;

Table gives test statistic of significant peak voxel(s) or, in case of no significance, of the overall peak voxel of the respective test. Voxel-level statistics were family-wise error corrected for the number of voxels within the search masks (whole brain or small-volume correction within the combined mask of dmPFC and right pSTS) for each test. Deviations from results reported in the main text are highlighted in bold. Significant whole-brain results are localized in MNI space and labeled according to the automated anatomical labeling atlas (aal). Abbreviations: TD typically developing, ASD autism spectrum disorder, pSTS posterior superior temporal sulcus, dmPFC dorsomedial prefrontal cortex, ROI region of interest, KCL Kings College London, UCBM University Campus Bio-Medico of Rome, UMCU University Medical Centre Utrecht, RUNMC Radboud University Nijmegen Medical Centre, CIMH Central Institute of Mental Health in Mannheim, UCAM University of Cambridge.


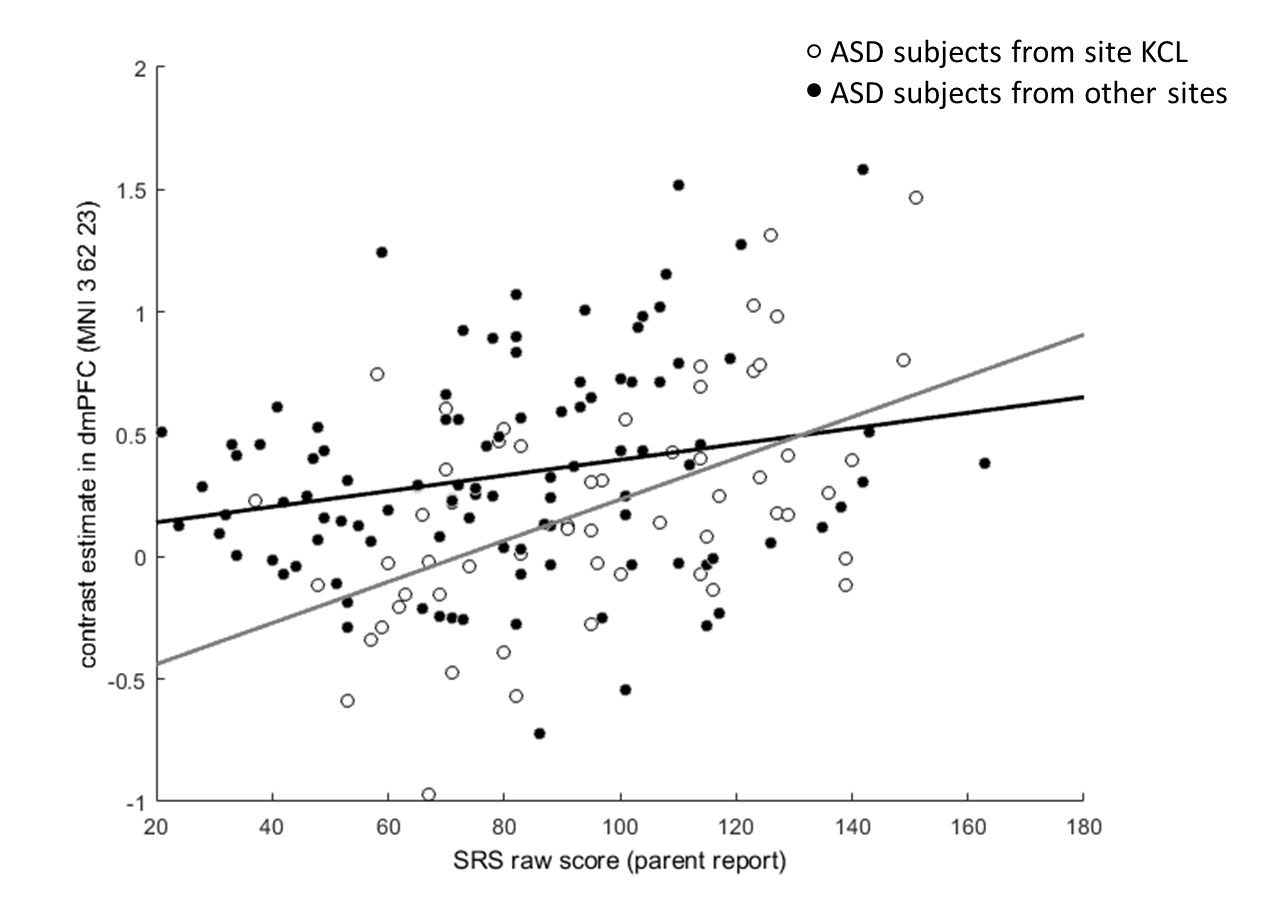


**Figure S5**: Scatter plots and linear model fits of the assocation between autism trait scores (parent-reported scores on the social responsiveness scale (SRS), x axis) and dmPFC activation (parametric modulation of levels of mental state attribution, y axis) in ASD subjects from site KCL (n=67) and ASD subjects recruited at other sites (n=138).

- 1. Behavioral performance

Behavioral performance was assessed as overall categorization accuracy (see main manuscript) and included as covariate of no interest in the sample-specific second-level GLMs on brain activation. Results do not suggest that individual differences in categorization accuracy affected autism-related effects across samples and search regions (whole brain, combined dmPFC and right pSTS ROI; table S10).

**Table S7**: Overview over effects of diagnosis (categorical) and autism traits (dimensional) on functional brain activation after controlling for behavioral performance.

|  | **TD vs. ASD (categorical)** | | | **positive association with autism traits (dimensional)** | | |
| --- | --- | --- | --- | --- | --- | --- |
|  | full sample | youth sample | adult sample | full sample (no TD adult^1^) | youth sample | adult sample (ASD only^1^) |
| original results (reported in main text) | no effect | no effect | no effect | positive association with inferior parietal cortex (whole brain) and dmPFC (ROI) | positive association with dmPFC (ROI) | no effect |
| whole-brain | no effect (all F_(1,383)_≤13.41, p_FWE_≥.734) | no effect (all F_(1,206)_≤12.96, p_FWE_≥.848) | no effect (all F_(1,167)_≤16.90, p_FWE_≥.415) | t_(235)=_4.80, p_FWE_=.021 at [54 -55 38] in inferior parietal cortex | no effect (all t_(165)_≤4.54, p_FWE_≥.067) | no effect (all t_(56)_≤4.60, p_FWE_≥.142) |
| right pSTS | no effect (all F_(1,383)_≤3.52, p_FWE_≥.919) | no effect (all F_(1,206)_≤7.96, p_FWE_≥.314) | no effect (all F_(1,167)_≤9.43, p_FWE_≥.214) | no effect (all t_(235)_≤2.90, p_FWE_≥.181) | no effect (all t_(165)_≤3.00, p_FWE_≥.143) | no effect (all t_(56)_≤2.01, p_FWE_≥.705) |
| dmPFC | no effect (all F_(1,383)_≤4.67, p_FWE_≥.785) | no effect (all F_(1,206)_≤7.94, p_FWE_≥.315) | no effect (all F_(1,267)_≤3.67, p_FWE_≥.937) | t_(235)=_3.53, p_FWE_=.034 at [3 62 23] | t_(165)=_3.78, p_FWE_=.016 at [3 56 26] | no effect (all t_(56)_≤2.91, p_FWE_≥.197) |

^1^ no parent-reported SRS-scores available for TD adults;

Table gives test statistic of significant peak voxel(s) or, in case of no significance, of the overall peak voxel of the respective test. Voxel-level statistics were family-wise error corrected for the number of voxels within the search masks (whole brain or small-volume correction within the combined mask of dmPFC and right pSTS) for each test. Deviations from results reported in the main text are highlighted in bold. Significant whole-brain results are localized in MNI space and labeled according to the automated anatomical labeling atlas (aal). Abbreviations: TD typically developing, ASD autism spectrum disorder, pSTS posterior superior temporal sulcus, dmPFC dorsomedial prefrontal cortex, ROI region of interest.

- 1. Medication

Medication use was confirmed for 81 individuals with ASD (adult: n=32, children: n=49) and 11 TD individuals (adult: n=2, children: n=9). Medication classes with most frequent use were psychostimulants and other drugs used to treat ADHD (39%), hypnotics/sedatives (34%), and antidepressants (25%). We performed three control analyses on the effect of medication.

1) Given the heterogeneity of drug classes, we used a simplistic approach and included a dichotomous covariate indicating medication use (yes/no) in the sample-specific second-level GLMs on brain activation. Results do not suggest an influence of medication on autism-related effects across samples and search regions (whole brain, combined dmPFC and right pSTS ROI; table S11).

**Table S8**: Overview over effects of diagnosis (categorical) and autism traits (dimensional) on functional brain activation after controlling for medication use.

|  | **TD vs. ASD (categorical)** | | | **positive association with autism traits (dimensional)** | | |
| --- | --- | --- | --- | --- | --- | --- |
|  | full sample | youth sample | adult sample | full sample (no TD adult^1^) | youth sample | adult sample (ASD only^1^) |
| original results (reported in main text) | no effect | no effect | no effect | positive association with inferior parietal cortex (whole brain) and dmPFC (ROI) | positive association with dmPFC (ROI) | no effect |
| whole-brain | no effect (all F_(1,383)_≤13.43, p_FWE_≥.729) | no effect (all F_(1,206)_≤18.97, p_FWE_≥.153) | no effect (all F_(1,167)_≤17.06, p_FWE_≥.395) | t_(235)=_4.82, p_FWE_=.021 at [54 -55 38] in inferior parietal cortex | no effect (all t_(165)_≤4.62, p_FWE_≥.053) | no effect (all t_(56)_≤4.12, p_FWE_≥.431) |
| right pSTS | no effect (all F_(1,383)_≤3.16, p_FWE_≥.946) | no effect (all F_(1,206)_≤10.17, p_FWE_≥.133) | no effect (all F_(1,167)_≤3.24, p_FWE_≥.963) | no effect (all t_(235)_≤2.87, p_FWE_≥.197) | no effect (all t_(165)_≤2.98, p_FWE_≥.152) | no effect (all t_(56)_≤1.86, p_FWE_≥.782) |
| dmPFC | no effect (all F_(1,383)_≤4.15, p_FWE_≥.853) | no effect (all F_(1,206)_≤7.73, p_FWE_≥.340) | no effect (all F_(1,167)_≤4.12, p_FWE_≥.900) | t_(235)=_3.50, p_FWE_=.037 at [3 62 23] | t_(165)=_3.74, p_FWE_=.018 at [3 56 26] | no effect (all t_(56)_≤2.70, p_FWE_≥.290) |

^1^ no parent-reported SRS-scores available for TD adults;

Table gives test statistic of significant peak voxel(s) or, in case of no significance, of the overall peak voxel of the respective test. Voxel-level statistics were family-wise error corrected for the number of voxels within the search masks (whole brain or small-volume correction within the combined mask of dmPFC and right pSTS) for each test. Deviations from results reported in the main text are highlighted in bold. Significant whole-brain results are localized in MNI space and labeled according to the automated anatomical labeling atlas (aal). Abbreviations: TD typically developing, ASD autism spectrum disorder, pSTS posterior superior temporal sulcus, dmPFC dorsomedial prefrontal cortex, ROI region of interest.

2) When excluding subjects with confirmed use of medication from the second-level analyses, the dimensional association in the dmPFC did not reach significance anymore (table S12). In contrast to the KCL site effect discussed above, this drop in significance cannot be attributed to differences in symptom severity between medicated and unmedicated ASD individuals (independent *t*-test on ADOS, ADI-R and parent-reported SRS score: all t<1.131, all p>.259).

**Table S9**: Overview over effects of diagnosis (categorical) and autism traits (dimensional) on functional brain activation after excluding individuals with confirmed use of medication (see table S1).

|  | **TD vs. ASD (categorical)** | | | **positive association with autism traits (dimensional)** | | |
| --- | --- | --- | --- | --- | --- | --- |
|  | full sample | youth sample | adult sample | full sample (no TD adult^1^) | youth sample | adult sample (ASD only^1^) |
| original results (reported in main text) | no effect | no effect | no effect | positive association with inferior parietal cortex (whole brain) and dmPFC (ROI) | positive association with dmPFC (ROI) | no effect |
| whole-brain | no effect (all F_(1,292)_≤12.72, p_FWE_≥.847) | **F_(1,149)_=23.98, p_FWE_=.044 at [48 5 17] in inferior frontal gyrus (pars opercularis)** | no effect (all F_(1,134)_≤14.02, p_FWE_≥.781) | **t_(167)=_4.59, p_FWE_=.044 at [-45 -37 8] in superior temporal gyrus** | no effect (all t_(122)_≤4.38, p_FWE_≥.103) | no effect (all t_(36)_≤4.26, p_FWE_≥.470) |
| right pSTS | no effect (all F_(1,292)_≤2.39, p_FWE_≥.983) | no effect (all F_(1,149)_≤8.93, p_FWE_≥.288) | no effect (all F_(1,134)_≤4.13, p_FWE_≥.877) | no effect (all t_(167)_≤2.22, p_FWE_≥.519) | no effect (all t_(122)_≤2.29, p_FWE_≥.467) | no effect (all t_(36)_≤1.76, p_FWE_≥.821) |
| dmPFC | no effect (all F_(1,292)_≤2.56, p_FWE_≥.978) | no effect (all F_(1,149)_≤8.43, p_FWE_≥.343) | no effect (all F_(1,134)_≤3.80, p_FWE_≥.910) | **no effect (all t_(167)_ ≤2.48, p_FWE_≥.174)** | **no effect (all t_(122)_ ≤3.14, p_FWE_≥.186)** | no effect (all t_(36)_≤3.45, p_FWE_≥.072) |

^1^ no parent-reported SRS-scores available for TD adults;

Table gives test statistic of significant peak voxel(s) or, in case of no significance, of the overall peak voxel of the respective test. Voxel-level statistics were family-wise error corrected for the number of voxels within the search masks (whole brain or small-volume correction within the combined mask of dmPFC and right pSTS) for each test. Deviations from results reported in the main text are highlighted in bold. Significant whole-brain results are localized in MNI space and labeled according to the automated anatomical labeling atlas (aal). Abbreviations: TD typically developing, ASD autism spectrum disorder, pSTS posterior superior temporal sulcus, dmPFC dorsomedial prefrontal cortex, ROI region of interest.

3) We also compared ASD individuals with and without medication. There was no effect of medication on brain activation during mentalizing in the full sample (whole brain: all F_(1,194)_≤18.73, p_FWE_≥.226; combined ROI mask: all F_(1,194)_≤5.57, p_FWE_≥.723) and the adult sample (whole brain: all F_(1,81)_≤11.85, p_FWE_≥.993; combined ROI mask: F_(1,81)_≤6.70, p_FWE_≥.555). In the youth sample, however, a whole-brain significant effect of medication emerged in the inferior frontal gyrus, extending into bilateral insula (contrast “unmedicated > medicated”: t_(102)_=5.18, p_FWE_=.009 at [57 5 11], t_(102)_=4.78, p_FWE_=.037 at [-33 17 2]; Figure S6), while no effect was observed in our key regions (all F_(1,102)_≤4.26, p_FWE_≥.880). The inspection of contrast estimates suggests that the medication effect was not driven by one of the three most prevalent medication classes in the youth sample (psychostimulants: 23%, hypnotics/sedatives: 21%, antipsychotics: 10%). Note, however, that among the medicated ASD subjects (n=49), 14 individuals were taking a combination of two medication classes (psychostimulants and hypnotics/sedatives: n=7; psychostimulants and antipsychotics: n=7). As a tentative interpretation, we propose that the medication might cause a relative attenuation of the brain’s salience and ventral attention system, with the key hub located in the anterior insula, and which might mediate improvements in distractibility, impulsivity, and levels of arousal (20).

However, we acknowledge that specific effects of medication need to be addressed in a more systematic way using appropriate samples.


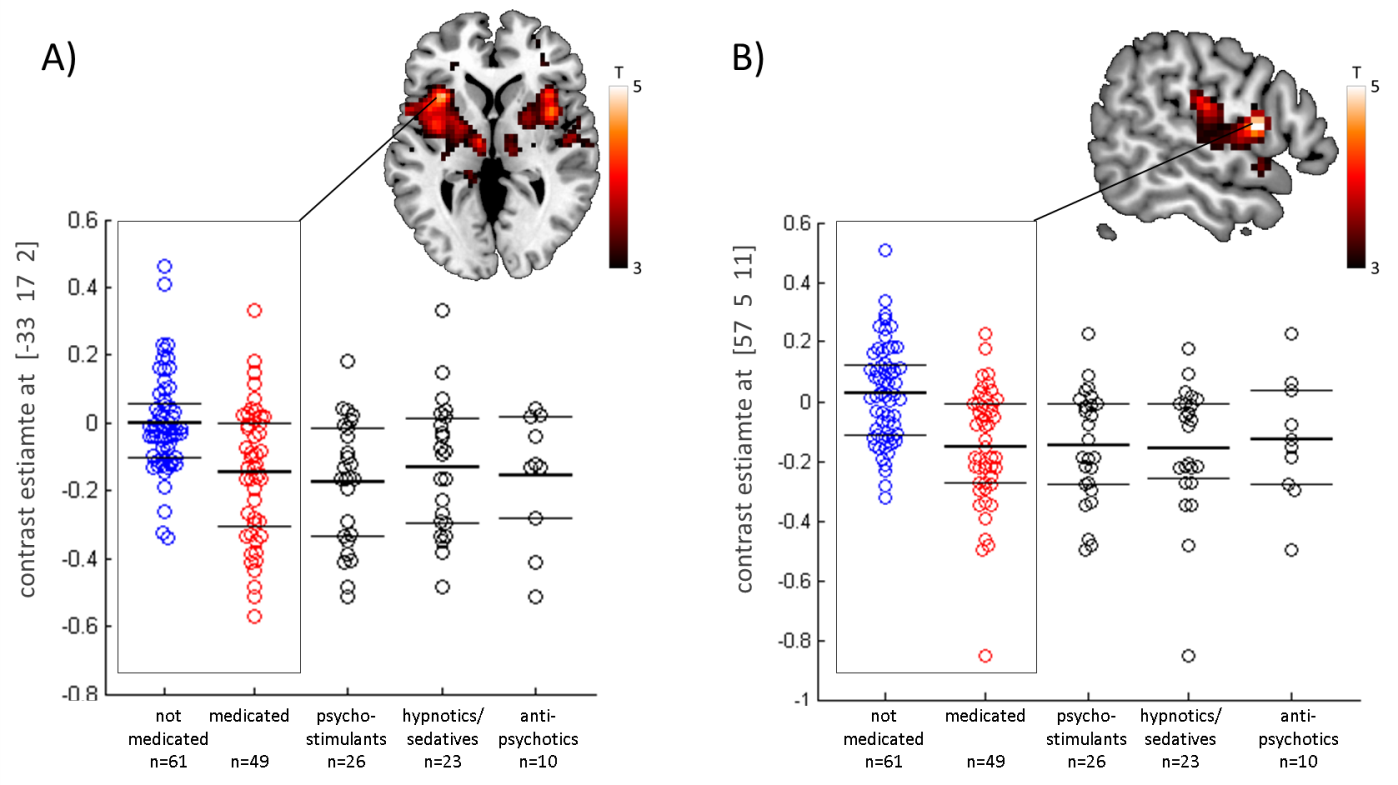


**Figure S6**: Brain sections illustrate the effect of medication in the left insula (A) and right inferior frontal gyrus (B) in children and adolescents with ASD (youth sample). Scatter plots demonstrate the distribution of contrast estimates in the identified peak voxels in ASD individuals with (red) and without (blue) medication, as well as in individuals taking a specific medication class (black). Mean values and inner quartiles (25th to 75th percentile) are indicated as thick and thin lines, respectively.

- 1. Motion and SNR

Data quality is heavily impacted by head motion (REF). As shown in section 2, individuals with ASD showed a trend-level higher amount of head motion (as assessed by mean framewise displacement (FD); (9)) than TD subjects (t_(392)_ = 1.877, *p* = .061).

Another measure of imaging data quality is signal-to-noise ratio (SNR), which was moderately correlated with head motion (Pearson r=.211, p<.001; Spearman rho=.170, p=.001).

In order to control for potential effects of data quality on our results, we included mean FD and SNR as covariates of no interest in the sample-specific second-level GLMs on brain activation. These analyses do not suggest that data quality had an impact on the results reported in the main manuscript (table S12).

**Table S10**: Overview over effects of diagnosis (categorical) and autism traits (dimensional) on functional brain activation after controlling for head motion and signal-to-noise ratio.

|  | **TD vs. ASD (categorical)** | | | **positive association with autism traits (dimensional)** | | |
| --- | --- | --- | --- | --- | --- | --- |
|  | full sample | youth sample | adult sample | full sample (no TD adult^1^) | youth sample | adult sample (ASD only^1^) |
| original results (reported in main text) | no effect | no effect | no effect | positive association with inferior parietal cortex (whole brain) and dmPFC (ROI) | positive association with dmPFC (ROI) | no effect |
| whole-brain | no effect (all F_(1,382)_≤12.62, p_FWE_≥.839) | no effect (all F_(1,205)_≤13.97, p_FWE_≥.711) | no effect (all F_(1,166)_≤17.77, p_FWE_≥.316) | t_(234)=_4.66, p_FWE_=.039 at [54 -55 38] in inferior parietal cortex | no effect (all F_(1,164)_≤4.61, p_FWE_≥.054) | no effect (all t_(55)_≤3.97, p_FWE_≥.581) |
| right pSTS | no effect (all F_(1,382)_≤3.75, p_FWE_≥.897) | no effect (all F_(1,205)_≤10.17, p_FWE_≥.133) | no effect (all F_(1,166)_≤8.56, p_FWE_≥.294) | no effect (all t_(234)_≤2.91, p_FWE_≥.179) | no effect (all t_(164)_≤2.98, p_FWE_≥.149) | no effect (all t_(55)_≤1.70, p_FWE_≥.852) |
| dmPFC | no effect (all F_(1,382)_≤4.04, p_FWE_≥.864) | no effect (all F_(1,205)_≤7.73, p_FWE_≥.340) | no effect (all F_(1,166)_≤3.48, p_FWE_≥.950) | t_(234)=_3.39, p_FWE_=.052 at [3 62 23] | t_(164)=_3.61, p_FWE_=.022 at [3 62 23] | no effect (all t_(55)_≤2.44, p_FWE_≥.437) |

^1^ no parent-reported SRS-scores available for TD adults;

Table gives test statistic of significant peak voxel(s) or, in case of no significance, of the overall peak voxel of the respective test. Voxel-level statistics were family-wise error corrected for the number of voxels within the search masks (whole brain or small-volume correction within the combined mask of dmPFC and right pSTS) for each test. Deviations from results reported in the main text are highlighted in bold. Significant whole-brain results are localized in MNI space and labeled according to the automated anatomical labeling atlas (aal). Abbreviations: TD typically developing, ASD autism spectrum disorder, pSTS posterior superior temporal sulcus, dmPFC dorsomedial prefrontal cortex, ROI region of interest.

- 1. Comorbidity (depression, anxiety, ADHD)

In the ASD sample, psychiatric comorbidities (except for psychosis or bipolar disorder) were allowed since up to 70% of people with ASD have one or more comorbidities (21), and since the presence of comorbidities reflects DSM-5 that allows co-occurring psychiatric disorders alongside an ASD diagnosis (22). These relatively lenient inclusion criteria were chosen to attain a sample representative of real-world ASD patient populations.

Here, we focus on ADHD, anxiety and depression as three common psychiatric comorbidities of ASD. In order to obtain dimensional scores of comorbidity across the full age range in TD and ASD subjects, the Development and Well Being Assessment (DAWBA; (4)) was used. DWABA is a package of questionnaires, interviews, and rating techniques designed to generate prediction scores for ICD-10 and DSM-IV psychiatric diagnoses. DAWBA scores reflect six levels of prediction (i.e. ~0.1%, ~0.5%, ~3%, ~15%, ~50%, >70%) of the probability of a disorder, ranging from very unlikely to probable. DAWBA scores are based on reports by the primary caregiver or the proband and show a good agreement with clinical ratings (4).

DAWBA scores were available for 77% (depression), 39% (ADHD) and 88% (anxiety) of TD individuals, and for 81% (depression), 68% (ADHD) and 89% (anxiety) of individuals with ASD. TD subjects had a mean DAWBA score of 0.3 ($\pm$0.7) for depression, 0.2 ($\pm$0.7) for ADHD, and 1.2($\pm$0.9) for anxiety. Individuals with ASD had a mean DAWBA score of 1.0 ($\pm$1.3) for depression, 1.6 ($\pm$1.6) for ADHD, and 2.5($\pm$1.3) for anxiety. When combining Levels 0-3 as ‘absent risk’ (i.e. having a risk score of ~0.5%, ~3%, ~15%) and Levels 4-5 as ‘present risk’ (~50%, >70%), the following numbers for ‘present risk’ can be derived for the TD and ASD samples: TD depression n=1, TD ADHD n=0, TD anxiety n=6, ASD depression n=10, ASD ADHD n=16, ASD anxiety n=51. Non-parametric correlation analyses in the full sample revealed medium-size correlations between autism traits (SRS raw scores, parent report) and DAWBA comorbidity risk scores for depression (spearman rho = .343, p<.001), ADHD (spearman rho = .573, p<.001) and anxiety (spearman rho = .572, p<.001).

In order to control for a potential impact of comorbidities on autism-related effects on functional activation, dimensional DAWBA risk scores for depression, ADHD and anxiety were included in the sample-specific second-level GLMs on brain activation. The shared variance between DAWBA comorbidity and autism trait scores is suggestive of an attenuation of the association between dmPFC activation and autism traits. Given the differences in DAWBA score availability, analyses were conducted separately for risk scores for depression, ADHD and anxiety, respectively.

The lack of a case-control difference on functional brain activation persisted when controlling for DAWBA comorbidity risk scores. As expected, the association between dmPFC activation and autism traits remained positive but dropped below the significance threshold when controlling for DAWBA scores for depression and anxiety in the full and youth samples. Interestingly, this effect was not observed when including DAWBA ADHD scores. The association between autism traits and activation in the inferior parietal cortex, which emerged in the whole-brain analyses in the full sample, was abolished when controlling for ADHD and anxiety (see tables S13-15).

These results suggest that the presence of comorbid symptoms of depression and anxiety led to a strengthening of the observed association between dmPFC activation and autism traits. ADHD symptoms, in contrast, did not influence the effect of autism traits in the dmPFC.

**Table S11:** Overview over effects of diagnosis (categorical) and autism traits (dimensional) on functional brain activation after controlling for DAWBA depression scores.

|  | **TD vs. ASD (categorical)** | | | **positive association with autism traits (dimensional)** | | |
| --- | --- | --- | --- | --- | --- | --- |
|  | full sample | youth sample | adult sample | full sample (no TD adult^1^) | youth sample | adult sample (ASD only^1^) |
| original results (reported in main text) | no effect | no effect | no effect | positive association with inferior parietal cortex (whole brain) and dmPFC (ROI) | positive association with dmPFC (ROI) | no effect |
| whole-brain | no effect (all F_(1,302)_≤15.28, p_FWE_≥.684) | no effect (all F_(1,167)_≤16.45, p_FWE_≥.544) | no effect (all F_(1,125)_≤16.84, p_FWE_≥.468) | t_(200)=_4.56, p_FWE_=.042 at [54 -55 38] in inferior parietal cortex | no effect (all t_(141)_≤3.87, p_FWE_≥.528) | **t_(45)=_3.34, p_FWE_=.027 at [39 -1 35] in right precentral gyrus** |
| right pSTS | no effect (all F_(1,302)_≤6.55, p_FWE_≥.616) | no effect (all F_(1,167)_≤9.81, p_FWE_≥.203) | no effect (all F_(1,125)_≤8.00, p_FWE_≥.364) | no effect (all t_(200)_≤3.08, p_FWE_≥.096) | no effect (all t_(141)_≤2.48, p_FWE_≥.420) | no effect (all t_(45)_≤2.28, p_FWE_≥.539) |
| dmPFC | no effect (all F_(1,302)_≤7.32, p_FWE_≥.496) | no effect (all F_(1,167)_≤6.86, p_FWE_≥.552) | no effect (all F_(1,125)_≤5.63, p_FWE_≥.706) | **no effect (all t_(200)_≤3.11, p_FWE_≥.088)** | **no effect (all t_(141)_≤3.07, p_FWE_≥.128)** | no effect (all t_(45)_≤2.09, p_FWE_≥.651) |

^1^ no parent-reported SRS-scores available for TD adults;

Table gives test statistic of significant peak voxel(s) or, in case of no significance, of the overall peak voxel of the respective test. Voxel-level statistics were family-wise error corrected for the number of voxels within the search masks (whole brain or small-volume correction within the combined mask of dmPFC and right pSTS) for each test. Deviations from results reported in the main text are highlighted in bold. Significant whole-brain results are localized in MNI space and labeled according to the automated anatomical labeling atlas (aal). Abbreviations: TD typically developing, ASD autism spectrum disorder, pSTS posterior superior temporal sulcus, dmPFC dorsomedial prefrontal cortex, ROI region of interest.

**Table S12:** Overview over effects of diagnosis (categorical) and autism traits (dimensional) on functional brain activation after controlling for DAWBA ADHD scores.

|  | **TD vs. ASD (categorical)** | | | **positive association with autism traits (dimensional)** | | |
| --- | --- | --- | --- | --- | --- | --- |
|  | full sample | youth sample | adult sample | full sample (no TD adult^1^) | youth sample | adult sample (ASD only^1^) |
| original results (reported in main text) | no effect | no effect | no effect | positive association with inferior parietal cortex (whole brain) and dmPFC (ROI) | positive association with dmPFC (ROI) | no effect |
| whole-brain | no effect (all F_(1,201)_≤13.49, p_FWE_≥.765) | no effect (all F_(1,147)_≤17.84, p_FWE_≥.378) | *no sufficient TD data* | **no effect (all t_(177)_≤4.33, p_FWE_≥.106)** | no effect (all t_(128)_≤4.06, p_FWE_≥.349) | no effect (all t_(39)_≤4.99, p_FWE_≥.096) |
| right pSTS | no effect (all F_(1,201)_≤6.00, p_FWE_≥.574) | no effect (all F_(1,147)_≤4.37, p_FWE_≥.897) | *no sufficient TD data* | **t_(177)=_3.50, p_FWE_=.032 at [57 -46 23])** | no effect (all t_(128)_≤2.53, p_FWE_≥.392) | no effect (all t_(39)_≤2.72, p_FWE_≥.302) |
| dmPFC | no effect (all F_(1,201)_≤5.26, p_FWE_≥.691) | no effect (all F_(1,147)_≤6.02, p_FWE_≥.682) | *no sufficient TD data* | t_(177)=_4.03, p_FWE_=.006 at [6 56 23] | t_(128)=_3.59, p_FWE_=.033 at [6 56 23] | no effect (all t_(39)_≤1.97, p_FWE_≥.732) |

^1^ no parent-reported SRS-scores available for TD adults;

Table gives test statistic of significant peak voxel(s) or, in case of no significance, of the overall peak voxel of the respective test. Voxel-level statistics were family-wise error corrected for the number of voxels within the search masks (whole brain or small-volume correction within the combined mask of dmPFC and right pSTS) for each test. Deviations from results reported in the main text are highlighted in bold. Significant whole-brain results are localized in MNI space and labeled according to the automated anatomical labeling atlas (aal). Abbreviations: TD typically developing, ASD autism spectrum disorder, pSTS posterior superior temporal sulcus, dmPFC dorsomedial prefrontal cortex, ROI region of interest.

**Table S13:** Overview over effects of diagnosis (categorical) and autism traits (dimensional) on functional brain activation after controlling for DAWBA anxiety scores.

| anxiety | **TD vs. ASD (categorical)** | | | **positive association with autism traits (dimensional)** | | |
| --- | --- | --- | --- | --- | --- | --- |
|  | full sample | youth sample | adult sample | full sample (no TD adult^1^) | youth sample | adult sample (ASD only^1^) |
| original results (reported in main text) | no effect | no effect | no effect | positive association with inferior parietal cortex (whole brain) and dmPFC (ROI) | positive association with dmPFC (ROI) | no effect |
| whole-brain | no effect (all F_(1,339)_≤12.67, p_FWE_≥.816) | no effect (all F_(1,178)_≤9.80, p_FWE_≥.999) | no effect (all F_(1,151)_≤14.18, p_FWE_≥.752) | **no effect (all t_(214)_≤4.09, p_FWE_≥.172)** | no effect (all t_(147)_≤3.65, p_FWE_≥.720) | no effect (all t_(53)_≤4.36, p_FWE_≥.269) |
| right pSTS | no effect (all F_(1,339)_≤2.48, p_FWE_≥.976) | no effect (all F_(1,178)_≤4.17, p_FWE_≥.856) | no effect (all F_(1,151)_≤6.83, p_FWE_≥.489) | no effect (all t_(214)_≤2.82, p_FWE_≥.166) | no effect (all t_(147)_≤2.79, p_FWE_≥.229) | no effect (all t_(53)_≤1.75, p_FWE_≥.828) |
| dmPFC | no effect (all F_(1,339)_≤4.31, p_FWE_≥.821) | no effect (all F_(1,178)_≤8.28, p_FWE_≥.282) | no effect (all F_(1,151)_≤5.13, p_FWE_≥.749) | **no effect (all t_(214)_≤3.07, p_FWE_≥.089)** | **no effect (all t_(147)_≤3.38, p_FWE_≥.054)** | no effect (all t_(53)_≤2.59, p_FWE_≥.347) |

^1^ no parent-reported SRS-scores available for TD adults;

Table gives test statistic of significant peak voxel(s) or, in case of no significance, of the overall peak voxel of the respective test. Voxel-level statistics were family-wise error corrected for the number of voxels within the search masks (whole brain or small-volume correction within the combined mask of dmPFC and right pSTS) for each test. Deviations from results reported in the main text are highlighted in bold. Significant whole-brain results are localized in MNI space and labeled according to the automated anatomical labeling atlas (aal). Abbreviations: TD typically developing, ASD autism spectrum disorder, pSTS posterior superior temporal sulcus, dmPFC dorsomedial prefrontal cortex, ROI region of interest.

- 1. Narrow ASD definition

Individuals with ASD were included in the LEAP cohort based on an existing clinical diagnosis (23) independent from current symptom load as assessed by Autism Diagnostic Observation Interview (ADOS;(2)) and the Autism Diagnostic Interview-Revised (ADI-R; (1)). The lack of case-control differences could therefore be a consequence of some LEAP volunteers not having strong ASD symptoms at the time of data assessment. In this control analysis, we restricted the analysis to individuals with ASD who met the threshold on both ADOS and ADI-R (following (24)). The proportion of individuals with ASD meeting this criterion was 49 % in the full sample, 52 % in the youth sample, and 45 % in the adult sample.

When restricting the sample-specific second-level GLM analyses to the narrow ASD definition, the lack of case-control differences on functional activation persisted (table S14). The association of autism traits with functional brain responses turned insignificant for the full sample. Interestingly, however, follow-up analyses outside brain space revealed an increase in effect size with preserved significance for the association between dmPFC activation and autism trait in this restricted sample (Figure S7; full sample including all ASD subjects (n=160), association at MNI coordinate [3 62 23]: Beta=.338, p=.001 (multiple regression, F(9,237)=3.206, p<.001, R2=.11); full sample including only ASD subjects with narrow ASD definition (n=85), association at MNI coordinate [3 62 23]: Beta=.450, p=.004 (multiple regression, F(8,160)=2.240, p=.027, R2=.10)).

These analyses therefore do not suggest a significant influence of the inclusion of individuals with ASD with rather low symptom load at the time of assessment.

**Table S14:** Overview over effects of diagnosis (categorical) and autism traits (dimensional) on functional brain activation, using the narrowly defined ASD subsample.

|  | **TD vs. ASD (categorical)** | | | **positive association with autism traits (dimensional)** | | |
| --- | --- | --- | --- | --- | --- | --- |
|  | full sample | youth sample | adult sample | full sample (no TD adult^1^) | youth sample | adult sample (ASD only^1^) |
| original results (reported in main text) | no effect | no effect | no effect | positive association with inferior parietal cortex (whole brain) and dmPFC (ROI) | positive association with dmPFC (ROI) | no effect |
| whole-brain | no effect (all F_(1,279)_≤16.17, p_FWE_≥.516) | no effect (all F_(1,154)_≤15.69, p_FWE_≥.489) | no effect (all F_(1,116)_≤15.13, p_FWE_≥.691) | **no effect (all t_(159)_≤4.08, p_FWE_≥.282)** | no effect (all t_(126)_≤4.59, p_FWE_≥.056) | no effect (all t_(22)_≤3.89, p_FWE_≥.913) |
| right pSTS | no effect (all F_(1,297)_≤5.89, p_FWE_≥.695) | no effect (all F_(1,154)_≤9.56, p_FWE_≥.170) | no effect (all F_(1,116)_≤6.64, p_FWE_≥.541) | no effect (all t_(159)_≤2.97, p_FWE_≥.150) | no effect (all t_(126)_≤2.43, p_FWE_≥.396) | no effect (all t_(22)_≤1.54, p_FWE_≥.887) |
| dmPFC | no effect (all F_(1,297)_≤4.48, p_FWE_≥.886) | no effect (all F_(1,154)_≤6.27, p_FWE_≥.536) | no effect (all F_(1,116)_≤3.27, p_FWE_≥.657) | **no effect (all t_(159)_≤3.09, p_FWE_≥.113)** | t_(126)=_3.53, p_FWE_=.031 at [-15 50 41] | no effect (all t_(22)_≤1.39, p_FWE_≥.920) |

^1^ no parent-reported SRS-scores available for TD adults;

Table gives test statistic of significant peak voxel(s) or, in case of no significance, of the overall peak voxel of the respective test. Voxel-level statistics were family-wise error corrected for the number of voxels within the search masks (whole brain or small-volume correction within the combined mask of dmPFC and right pSTS) for each test. Deviations from results reported in the main text are highlighted in bold. Significant whole-brain results are localized in MNI space and labeled according to the automated anatomical labeling atlas (aal). Abbreviations: TD typically developing, ASD autism spectrum disorder, pSTS posterior superior temporal sulcus, dmPFC dorsomedial prefrontal cortex, ROI region of interest.


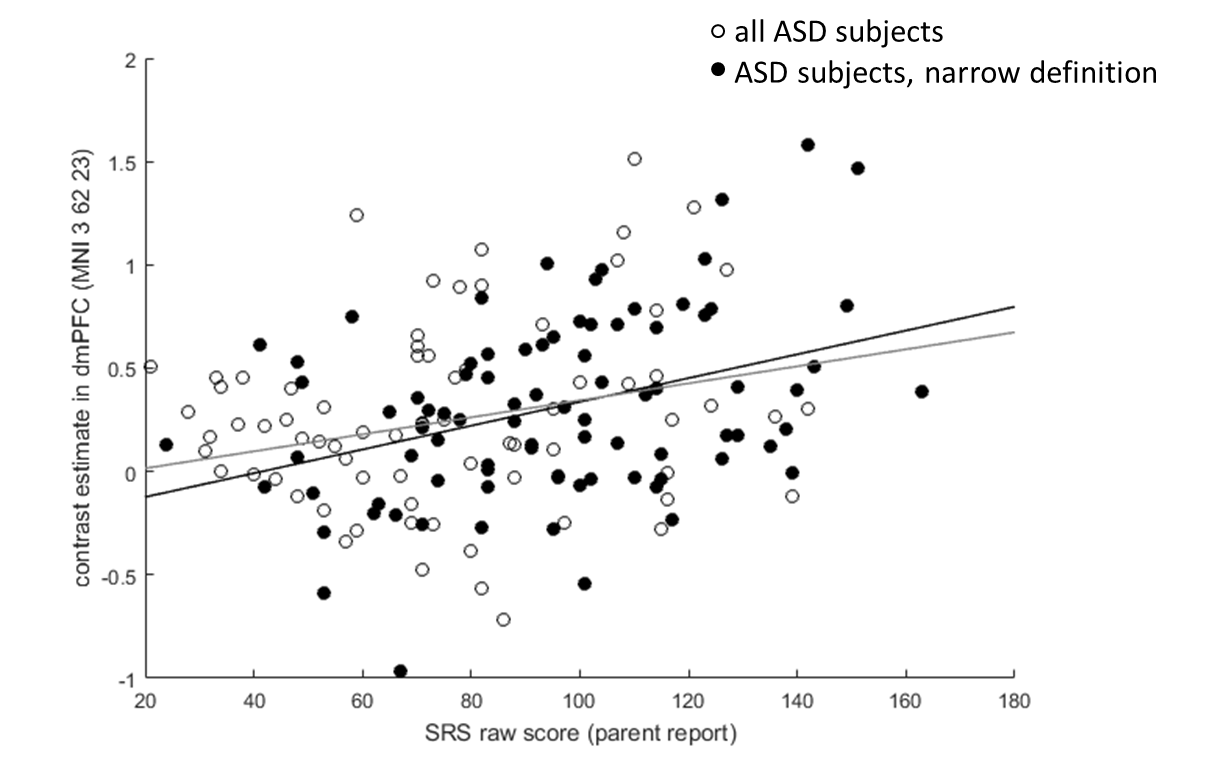


**Figure S7**: Scatter plots and linear model fits of the assocation between autism trait scores (parent-reported scores on the social responsiveness scale (SRS), x axis) and dmPFC activation (parametric modulation by levels of mental state attribution, y axis) in the full ASD sample (n=205) and in the narrowly defined ASD subsample (n=100).

- 1. SRS self-report

Depending on the recruitment schedule, autism traits were assessed as parent-reported SRS scores (assessed in the youth sample and in ASD adults), and/or self-reported SRS scores (assessed in the adult sample and in TD adolescents aged 12 to 18 years). Since parent- and self-reported scores show a certain degree of deviation despite significant correlation (Pearson correlation r=.663, p<.001, n=160), we did not mix both informant types in the main analyses, and thus excluded adult TD subjects from the dimensional analysis. Here, we repeated the dimensional analysis using self-reported instead of parent-reported SRS scores, which included all adult subjects as well as TD adolescents.

These analyses did not reveal any associations between functional brain responses and self-reported autism traits, which might be due to the informant type, but also to sample characteristics (e.g. older individuals, low proportion of individuals with ASD). The latter is certainly an important reason since the association of functional activation with parent-reported SRS scores was clearly driven by individuals with ASD, in particular within the children age, who were not included in the present control analysis.

**Table S15:** Overview over the association between self-reported SRS scores and functional brain activation.

|  | **positive association with autism traits (dimensional)** | |
| --- | --- | --- |
|  | adult sample | full sample: adults and TD adolescents (12-18 years) |
|  | main analysis: no effect | ---- |
| whole-brain | no effect (all t_(134)_≤3.52, p_FWE_≥.847) | no effect (all t_(244)_≤3.43, p_FWE_≥.872) |
| right pSTS | no effect (all t_(244)_≤1.16, p_FWE_≥.872) | no effect (all t_(244)_≤1.98, p_FWE_≥.724) |
| dmPFC | no effect (all t_(134)_≤2.24, p_FWE_≥.972) | no effect (all t_(244)_≤1.66, p_FWE_≥.876) |

^1^ no parent-reported SRS-scores available for TD adults;

Table gives test statistic of significant peak voxel(s) or, in case of no significance, of the overall peak voxel of the respective test. Voxel-level statistics were family-wise error corrected for the number of voxels within the search masks (whole brain or small-volume correction within the combined mask of dmPFC and right pSTS) for each test. Abbreviations: TD typically developing, pSTS posterior superior temporal sulcus, dmPFC dorsomedial prefrontal cortex.

1. Effect of sex

While we controlled for the effect of sex in all analyses, we additionally explored whether ASD-related effects on brain activation during on-line mentalizing were conditional to the participant’s sex (diagnosis x sex interaction).

We did not observe a differential effect of sex on brain activation between cases and controls, neither on the whole-brain level nor within regions of interest (full sample, right pSTS: *F* ≤ 5.15, *p*_FWE_ (small volume corrected, SVC) ≥ .711, dmPFC: *F* ≤ 4.16, *p*_FWE_ (SVC) ≥ .851; youth sample, right pSTS: *F* ≤ 4.66, *p*_FWE_ (SVC) ≥ .791, dmPFC: *F* ≤ 4.55, *p*_FWE_ (SVC) ≥ .806; adult sample, right pSTS: *F* ≤ 5.23, *p*_FWE_ (SVC) ≥ .765, dmPFC: *F* ≤ 3.17, *p*_FWE_ (SVC) ≥ .967).

We also did not observe a differential effect of sex on the association between functional activation and autistic traits as assessed by parent-reported SRS-2 scores (full sample, right pSTS: *F* ≤ 5.53, *p*_FWE_ (SVC) ≥ .688, dmPFC: *F* ≤ 10.55, *p*_FWE_ (SVC) ≥ .122; youth sample, right pSTS: *F* ≤ 8.42, *p*_FWE_ (SVC) ≥ .279, dmPFC: *F* ≤ 7.88, *p*_FWE_ (SVC) ≥ .338; adult sample, right pSTS: *F* ≤ 6.05, *p*_FWE_ (SVC) ≥ .592, dmPFC: *F* ≤ 6.60, *p*_FWE_ (SVC) ≥ .592).

These results suggest that sex did not modulate ASD-related effects on functional responses to the animated shapes.

1. Association between video categorization and dmPFC activation

We followed up on the significant association between autism traits and differential dmPFC activation.

First, in order to test whether dmPFC activation correlated with perceived mentalizing demands, we used the participant’s own categorization scores (instead of the predefined categorization scores) as parametric modulator in the first-level model. Whole-brain effects of parametric modulation were highly comparable between both versions. Activation in the dmPFC was robustly modulated by perceived mentalizing demand (*t* = 12.51, p*_FWE_* = <.001, whole brain corrected at MNI x = -6, y = 53, z = 32) which was not affected by diagnostic status (F ≤ 1.55, *p*_FWE_ (SVC_dmPFC_) ≥ .924, small volume corrected within the mask of the dmPFC with n = 280 voxels) or age (F ≤ 5.79, *p*_FWE_ (SVC_dmPFC_) ≥ .349). Similarly, The association with autistic traits (SRS-2 scores) persisted at *t* = 3.41, *p*_FWE_ (SVC_dmPFC_) = .029, small volume corrected at MNI x = 3, y = 56, z = 26).

Second, we tested whether dmPFC activation was affected by interindividual differences in ToM categorization accuracy. We included accuracy scores as a covariate of interest in the general linear model. Results do not support the hypothesis of an effect of ToM categorization accuracy on dmPFC activation (F ≤ 8.60, *p*_FWE_ (SVC_dmPFC_) ≥ .136).

Third, we tested whether the impact of autistic traits on dmPFC activation was moderated by ToM categorization accuracy (i.e. interaction between autistic traits and accuracy scores). This was not the case (F ≤ 4.28, *p*_FWE_ (SVC_dmPFC_) ≥ .717).

1. References

1. Rutter M, Le Couteur A, Lord C. Autism Diagnostic Interview-Revised. Los Angeles: Western Psychological Services; 2003.

2. Lord C, Risi S, Lambrecht L, Cook EH, Jr., Leventhal BL, DiLavore PC, et al. The autism diagnostic observation schedule-generic: a standard measure of social and communication deficits associated with the spectrum of autism. Journal of autism and developmental disorders. 2000;30(3):205-23.

3. Constantino J, Gruber C. Social Responsiveness Scale. In: Volkmar FR, editor. Encyclopedia of Autism Spectrum Disorders. New York, NY: Springer; 2013.

4. Goodman A, Heiervang E, Collishaw S, Goodman R. The 'DAWBA bands' as an ordered-categorical measure of child mental health: description and validation in British and Norwegian samples. Soc Psychiatry Psychiatr Epidemiol. 2011;46(6):521-32.

5. Loth E, Charman T, Mason L, Tillmann J, Jones EJH, Wooldridge C, et al. The EU-AIMS Longitudinal European Autism Project (LEAP): design and methodologies to identify and validate stratification biomarkers for autism spectrum disorders. Molecular autism. 2017;8:24.

6. Moessnang C, Schafer A, Bilek E, Roux P, Otto K, Baumeister S, et al. Specificity, reliability and sensitivity of social brain responses during spontaneous mentalizing. Social cognitive and affective neuroscience. 2016.

7. Plichta MM, Grimm O, Morgen K, Mier D, Sauer C, Haddad L, et al. Amygdala habituation: A reliable fMRI phenotype. NeuroImage. 2014;103C:383-90.

8. Plichta MM, Schwarz AJ, Grimm O, Morgen K, Mier D, Haddad L, et al. Test-retest reliability of evoked BOLD signals from a cognitive-emotive fMRI test battery. NeuroImage. 2012;60(3):1746-58.

9. Jenkinson M, Bannister P, Brady M, Smith S. Improved optimization for the robust and accurate linear registration and motion correction of brain images. NeuroImage. 2002;17(2):825-41.

10. Power JD, Barnes KA, Snyder AZ, Schlaggar BL, Petersen SE. Spurious but systematic correlations in functional connectivity MRI networks arise from subject motion. NeuroImage. 2012;59(3):2142-54.

11. Castelli F, Frith C, Happe F, Frith U. Autism, Asperger syndrome and brain mechanisms for the attribution of mental states to animated shapes. Brain : a journal of neurology. 2002;125(Pt 8):1839-49.

12. Abell F, Happé F, Frith U. Do triangles play tricks? Attribution of mental states to animated shapes in normal and abnormal development. Cognitive Development. 2000;15:1-16.

13. White SJ, Coniston D, Rogers R, Frith U. Developing the Frith-Happe animations: a quick and objective test of Theory of Mind for adults with autism. Autism research : official journal of the International Society for Autism Research. 2011;4(2):149-54.

14. Moessnang C, Otto K, Bilek E, Schafer A, Baumeister S, Hohmann S, et al. Differential responses of the dorsomedial prefrontal cortex and right posterior superior temporal sulcus to spontaneous mentalizing. Human brain mapping. 2017;38(8):3791-803.

15. Wechsler D. Wechsler Abbreviated Scale of Intelligence-Second Edition (WASI-II). San Antonio: NCS Pearson; 2011.

16. Wechsler D. Wechsler Intelligence Scale for Children - Third Edition. San Antonio: Psychological Corporation; 1991.

17. Wechsler D. Wechsler Intellligence Scale for Children - Fourth Edition. San Antonio: Psychological Corporation; 2003.

18. Wechsler D. Wechsler Adult Intelligence Scale - Third Edition. San Antonio: The Psychological Corporation; 1997.

19. Wechsler D. Wechsler Adult Intelligence Scale - Fourth Edition. San Antonio: Pearson; 2008.

20. Uddin LQ. Salience processing and insular cortical function and dysfunction. Nature reviews Neuroscience. 2015;16(1):55-61.

21. Simonoff E, Pickles A, Charman T, Chandler S, Loucas T, Baird G. Psychiatric disorders in children with autism spectrum disorders: prevalence, comorbidity, and associated factors in a population-derived sample. J Am Acad Child Adolesc Psychiatry. 2008;47(8):921-9.

22. Association AP. Diagnoistc and Statistical Manual of Mental Disorders. 5 ed. Washington DC: American Psychiatric Association; 2013.

23. Charman T, Loth E, Tillmann J, Crawley D, Wooldridge C, Goyard D, et al. The EU-AIMS Longitudinal European Autism Project (LEAP): clinical characterisation. Molecular autism. 2017;8:27.

24. Risi S, Lord C, Gotham K, Corsello C, Chrysler C, Szatmari P, et al. Combining information from multiple sources in the diagnosis of autism spectrum disorders. J Am Acad Child Adolesc Psychiatry. 2006;45(9):1094-103.
